# PyroTRF-ID: a novel bioinformatics methodology for the affiliation of terminal-restriction fragments using 16S rRNA gene pyrosequencing data

**DOI:** 10.1186/1471-2180-12-306

**Published:** 2012-12-27

**Authors:** David G Weissbrodt, Noam Shani, Lucas Sinclair, Grégory Lefebvre, Pierre Rossi, Julien Maillard, Jacques Rougemont, Christof Holliger

**Affiliations:** 1 Ecole Polytechnique Fédérale de Lausanne, School of Architecture, Civil and Environmental Engineering, Laboratory for Environmental Biotechnology, Station 6, Lausanne, 1015, Switzerland; 2 Ecole Polytechnique Fédérale de Lausanne, School of Life Sciences, Bioinformatics and Biostatistics Core Facility, Lausanne, Switzerland; 3 Swiss Institute of Bioinformatics, Lausanne, Switzerland; 4 Ecole Polytechnique Fédérale de Lausanne, School of Architecture, Civil and Environmental Engineering, Central Environmental Molecular Biology Laboratory, Lausanne, Switzerland; 5 Uppsala University, Limnology Department, Evolutionary Biology Centre, Uppsala, Sweden; 6 Nestlé Institute of Health Sciences, Lausanne, Switzerland

**Keywords:** Microbial ecology, T-RFLP, Pyrosequencing, Digital T-RFLP, Bioinformatics methodology

## Abstract

**Background:**

In molecular microbial ecology, massive sequencing is gradually replacing classical fingerprinting techniques such as terminal-restriction fragment length polymorphism (T-RFLP) combined with cloning-sequencing for the characterization of microbiomes. Here, a bioinformatics methodology for pyrosequencing-based T-RF identification (PyroTRF-ID) was developed to combine pyrosequencing and T-RFLP approaches for the description of microbial communities. The strength of this methodology relies on the identification of T-RFs by comparison of experimental and digital T-RFLP profiles obtained from the same samples. DNA extracts were subjected to amplification of the 16S rRNA gene pool, T-RFLP with the *Hae*III restriction enzyme, 454 tag encoded FLX amplicon pyrosequencing, and PyroTRF-ID analysis. Digital T-RFLP profiles were generated from the denoised full pyrosequencing datasets, and the sequences contributing to each digital T-RF were classified to taxonomic bins using the Greengenes reference database. The method was tested both on bacterial communities found in chloroethene-contaminated groundwater samples and in aerobic granular sludge biofilms originating from wastewater treatment systems.

**Results:**

PyroTRF-ID was efficient for high-throughput mapping and digital T-RFLP profiling of pyrosequencing datasets. After denoising, a dataset comprising ca. 10′000 reads of 300 to 500 bp was typically processed within ca. 20 minutes on a high-performance computing cluster, running on a Linux-related CentOS 5.5 operating system, enabling parallel processing of multiple samples. Both digital and experimental T-RFLP profiles were aligned with maximum cross-correlation coefficients of 0.71 and 0.92 for high- and low-complexity environments, respectively. On average, 63±18% of all experimental T-RFs (30 to 93 peaks per sample) were affiliated to phylotypes.

**Conclusions:**

PyroTRF-ID profits from complementary advantages of pyrosequencing and T-RFLP and is particularly adapted for optimizing laboratory and computational efforts to describe microbial communities and their dynamics in any biological system. The high resolution of the microbial community composition is provided by pyrosequencing, which can be performed on a restricted set of selected samples, whereas T-RFLP enables simultaneous fingerprinting of numerous samples at relatively low cost and is especially adapted for routine analysis and follow-up of microbial communities on the long run.

## Background

Molecular microbial ecology has become an important discipline in natural and medical sciences. Research on the structure, dynamics and evolution of microbial communities in environmental, human, and engineered systems provides substantial scientific knowledge for understanding the underlying microbial processes, for predicting their behavior, and for controlling, favoring, or suppressing target populations [1,2].

Different analytical methods have been successively developed for the assessment of microbial communities via profiling or metagenomic approaches [3]. Terminal-restriction fragment length polymorphism (T-RFLP) analysis has been widely used over the last decade for culture-independent assessment of complex microbial community structures [4,5]. Standardized, robust, and highly reproducible T-RFLP has become the method of choice for community fingerprinting since its automation in capillary electrophoresis devices has enabled the simultaneous analysis of numerous samples at relatively low cost [6-8]. Cloning and sequencing methods have been optimized in parallel for taxonomic affiliation of terminal-restriction fragments (T-RF) [9,10]. This approach however remains time-consuming and often leads to only partial characterization of the apparent microbial diversity [11]. On the other hand, next-generation sequencing (NGS) technologies have recently been applied for comprehensive high-throughput analyses of microbiomes with reduced sequencing costs [12-16] and high reproducibility [17]. Metagenomics projects have however generated novel requirements in resource and expertise for generating, processing, and interpreting large datasets [18-23]. Overall, ′omics′ technologies challenge the field of bioinformatics to design tailored computing solutions for enhanced production of scientific knowledge from massive datasets. While NGS techniques tend to progressively replace the traditional combination of T-RFLP and cloning-sequencing, recent studies have demonstrated the benefits of using both techniques to complement each other [24-28]. The combination of routine T-RFLP and NGS strategies could offer an efficient trade-off between laboratory efforts required for the in-depth analysis of bacterial communities and the financial and infrastructural costs related to datasets processing.

If T-RFLP and NGS are meant to be used concomitantly for the investigation of microbial systems, one key objective is to link T-RFs to phylotypes. In parallel to early developments of T-RFLP methods, several computational procedures have been proposed to predict T-RF sizes and to phylogenetically affiliate T-RFs. For instance, TAP T-RFLP [29], TRiFLe [30] and T-RFPred [31] have been developed to perform *in silico* digestion of datasets of 16S rRNA gene sequences, originating mostly from clone libraries or reference public databases. REPK [25] has been designed to screen for single and combinations of restriction enzymes for the optimization of T-RFLP profiles, and to design experimental strategies. All these programs do not involve comparison of *in silico* profiles with experimental data.

In the current study, we propose a novel bioinformatics methodology, called PyroTRF-ID, to assign phylogenetic affiliations to experimental T-RFs by coupling pyrosequencing and T-RFLP datasets obtained from the same biological samples. A recent study showing that natural bacterial community structures analyzed with both techniques were very similar [17] strengthened the here adopted conceptual approach. The methodological objectives were to generate digital T-RFLP (dT-RFLP) profiles from full pyrosequencing datasets, to cross-correlate them to the experimental T-RFLP (eT-RFLP) profiles, and to affiliate eT-RFs to closest bacterial relatives, in a fully automated procedure. The effects of different processing algorithms are discussed. An additional functionality was developed to assess the impact of restriction enzymes on resolution and representativeness of T-RFLP profiles. Validation was conducted with high- and low-complexity bacterial communities. This dual methodology was meant to process single DNA extracts in T-RFLP and pyrosequencing with similar PCR conditions, and therefore aimed to preserve the original microbial complexity of the investigated samples.

## Methods

### Samples

Two different biological systems were used for analytical procedure validation. The first set comprised ten groundwater (GRW) samples from two different chloroethene-contaminated aquifers that have been previously described by Aeppli et al. [32] and Shani [33]. The second set consisted of five aerobic granular sludge (AGS) biofilm samples from anaerobic-aerobic sequencing batch reactors operated for full biological nutrient removal from an acetate-based synthetic wastewater. The AGS system has been described previously [34] and displayed a lower bacterial community complexity (richness of 42±6 eT-RFs, Shannon′s H′ diversity of 2.5±0.2) than the GRW samples (richness of 67±15 eT-RFs, Shannon′s H′ diversity of 3.3±0.5).

### DNA extraction

GRW samples were filtered through 0.2-μm autoclaved polycarbonate membranes (Isopore™ Membrane Filters, Millipore) with a mobile filtration system (Filter Funnel Manifolds, Pall Corporation). DNA was extracted using the PowerSoil™ DNA Extraction Kit (Mo-Bio Laboratories, Inc.) following the manufacturer instructions, except that the samples were processed in a bead-beater (Fastprep FP120, Bio101) at 4.5 m·s^-1^ for 30 s after the addition of solution C1.

DNA from AGS samples was extracted with the automated Maxwell 16 Tissue DNA Purification System (Promega, Duebendorf, Switzerland) according to manufacturer′s instructions with following modifications. An aliquot of 100 mg of ground granular sludge was preliminarily digested during 1 h at 37°C in 500 μL of a solution composed of 5 mg·mL^-1^ lysozyme in TE buffer (10 mM Tris–HCl, 0.1 mM EDTA, pH 7.5). The DNA extracts were resuspended in 300 μL of TE buffer.

All extracted DNA samples were quantified with the ND-1000 Nanodrop® spectrophotometer (Thermo Fisher Scientific, USA) and stored at −20°C until analysis.

### Experimental T-RFLP

The eT-RFLP analysis of the GRW series was done according to Rossi et al. [8] with following modifications: (i) 30 μL PCR reactions contained 3 μL 10× Y buffer, 2.4 μL 10 mM dNTPs, 1.5 μL of each primer at 10 μM, 6 μL 5× enhancer P solution, 1.5 U PeqGold Taq polymerase (Peqlab), and 0.2 ng·μL^-1^ template DNA (final concentration), completed with autoclaved and UV-treated Milli-Q water (Millipore, USA); (ii) for each DNA extract, PCR amplification was carried out in triplicate. Samples from the AGS series were analyzed by eT-RFLP according to Ebrahimi et al. [35] with following modifications: (i) Go Taq polymerase (Promega, Switzerland) was used for PCR amplification; (ii) forward primer was FAM-labeled; (iii) the PCR program was modified to increase the initial denaturation to 10 min, the cycle denaturation step to 1 min, and 30 cycles of amplification. All PCRs were carried out using the labeled forward primer 8f (FAM-5′-AGAGTTTGATCMTGGCTCAG-3′) and the reverse primer 518r (5′-ATTACCGCGGCTGCTGG-3′). For details, refer to Weissbrodt et al. [34].

The resulting eT-RFLP profiles were generated between 50 and 500 bp as described in [8]. The eT-RFLP profiles were aligned using the Treeflap crosstab macro [36] and expressed as relative contributions of operational taxonomic units (OTUs). For GRW samples which exhibited numerous low abundant OTUs, the final bacterial community datasets were constructed as follows: multivariate Ruzicka dissimilarities were computed between replicates of eT-RFLP profiles with R [37] and the additional package Vegan [38]; the profile at the centroid (i.e. displaying the lowest dissimilarity with its replicates) was selected for each sample to build the final community profiles. For AGS samples which were characterized by less complex communities, triplicates were periodically measured and resulted in a mean relative standard coefficient of 6% over the analytical method.

### Cloning and sequencing

Clone libraries were constructed with the 16S rRNA gene pool amplified from DNA samples using the same PCR procedures as described in the eT-RFLP method but with an unlabeled 8f primer. The PCR products were purified with the purification kit Montage® PCR Centrifugal Filter Devices (Millipore, USA), ligated into pGEM®-T Easy vector (Promega, USA) and transformed into *E. coli* XL1-Blue competent cells (Agilent Technologies, USA). The eT-RFLP procedure was then applied on isolated colonies in order to screen for the dominant eT-RFs obtained previously by eT-RFLP on the entire 16S rRNA gene pool. Then the 16S rRNA gene was amplified from selected colonies using PCR with primers T7 and SP6 (Promega, USA) and purified as described above. A sequencing reaction was carried out on each purified PCR product as described in [39]. Sequences were aligned in BioEdit [40], and primer sequences were removed. Sequences were analyzed for chimeras using Bellerophon [41], and dT-RFs of selected clones were produced by *in silico* digestion using TRiFLe [30] for comparison with eT-RFs.

### Pyrosequencing

A total of 15 biological samples were analyzed using bacterial tag encoded FLX amplicon pyrosequencing analysis. A first set of DNA extracts from GRW and AGS samples were sent for sequencing to Research and Testing Laboratory LLC (Lubbock, TX, USA). The samples underwent partial amplification of the V1-V3 region of the 16S rRNA gene by PCR with unlabeled 8f and 518r primers, secondary PCR with tagged fusion primers for FLX amplicon sequencing, emulsion-based clonal amplification (emPCR), and GS FLX sequencing targeting at least 3′000 reads with the 454 GS-FLX Titanium Genome Sequencing System technology (Roche, Switzerland). The whole sample preparation protocol has been made available by the company in the publication of Sun et al. [13]. This series refers, in the present study, to the low reads amount pyrosequencing procedure (LowRA). The DNA extract of one AGS sample was analyzed in triplicate through the whole analytical method from pyrosequencing (LowRA) to PyroTRF-ID analysis.

A second set of amplicons from different GRW samples was analyzed by GATC Biotech AG (Konstanz, Germany) following an analog procedure but targeting at least 10′000 reads (referred to as the high reads amount method, HighRA, hereafter). The A- and B-adapters for sequencing with the Roche technology were ligated to the ends of the DNA fragments. The samples were run on a 2% agarose gel with TAE buffer and the band in a size range of 700–900 bp, 450–650 bp, or 100–500 bp, respectively, was excised and column purified. After concentration measurement the differently tagged libraries were pooled. The three resulting library pools were immobilized onto DNA capture beads and the amplicon-beads obtained were amplified through emPCR according to the manufacturer′s recommendations. Following amplification, the emulsion was chemically broken and the beads carrying the amplified DNA library were recovered and washed by filtration. Each pool was sequenced on a quarter GS FLX Pico-Titer plate device with GS FLX Titanium XLR70 chemistry on a GS FLX+ Instrument. The GS FLX System Software Version 2.6 was used and the GS FLX produced the sequence data as Standard Flowgram Format (SFF) file containing flowgrams for each read with basecalls and per-base quality scores.

### Development of the PyroTRF-ID bioinformatics methodology

**Figure 1:**
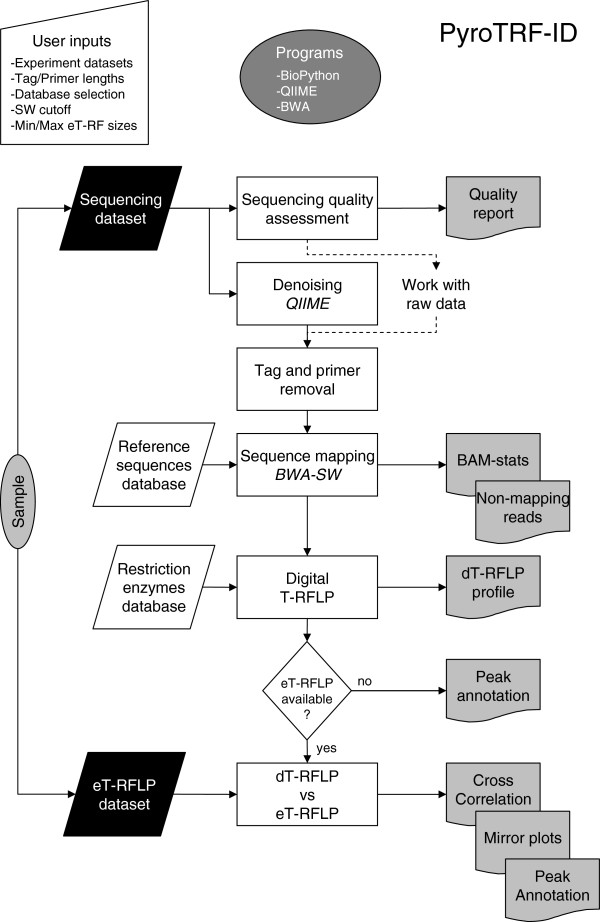
**Data workflow in the PyroTRF-ID bioinformatics methodology.** Experimental pyrosequencing and T-RFLP input datasets (*black parallelograms*), reference input databases (*white parallelograms*), data processing (*white rectangles*), output files (*grey sheets*).

The PyroTRF-ID bioinformatics methodology for identification of T-RFs from pyrosequencing datasets was coded in Python for compatibility with the *BioLinux* open software strategy [42]. PyroTRF-ID runs were run on the Vital-IT high performance computing center (HPCC) of the Swiss Institute of Bioinformatics (Switzerland). All documentation needed for implementing the methodology is available at http://bbcf.epfl.ch/PyroTRF-ID/. The flowchart description of PyroTRF-ID is depicted in Figure 1, and computational parameters are described hereafter.

#### Input files

Input 454 tag-encoded pyrosequencing datasets were used either in raw standard flowgram (.sff), or as pre-denoised fasta format (.fasta) as presented below. Input eT-RFLP datasets were provided in coma-separated-values format (.csv).

#### Denoising

Sequence denoising was integrated in the PyroTRF-ID workflow but this feature can be disabled by the user. It requires the independent installation of the QIIME software [43] to decompose and denoise the .sff files containing the whole pyrosequencing information into .sff.txt, .fasta and .qual files. Briefly, the script *split_libraries.py* was used first to remove tags and primers. Sequences were then filtered based on two criteria: (i) a sequence length ranging from the minimum (default value of 300 bp) and maximum 500-bp amplicon length, and (ii) a PHRED sequencing quality score above 20 according to Ewing and Green [44]. Denoising for the removal of classical 454 pyrosequencing flowgram errors such as homopolymers [45,46] was carried out with the script *denoise_wrapper.py*. Denoised sequences were processed using the script *inflate_denoiser_output.py* in order to generate clusters of sequences with at least 97% identity as conventionally used in the microbial ecology community [47]. Based on computation of statistical distance matrices, one representative sequence (centroid) was selected for each cluster. With this procedure, a new file was created containing cluster centroids inflated according to the original cluster sizes as well as non-clustering sequences (singletons). The denoising step on the HPCC typically lasted approximately 13 h and 5 h for HighRA and LowRA datasets, respectively.

#### Mapping

Mapping of sequences was performed using the Burrows-Wheeler Aligner′s Smith-Waterman (BWA-SW) alignment algorithm [48] against the Greengenes database [49]. The SW score was used as mapping quality criterion [50,51]. It can be set by the user according to research needs. Sequences with SW scores below 150 were removed from the pipeline. SW cutoffs have typically been used in a range between 100 and 250 [52,53]. This score can be adapted to reduce the probability of mismatches. SW scores normalized by sequence length were computed to allow comparison between sequences of various lengths. Two files were generated consecutive to mapping. The first one provided general mapping statistics for each sample. The second one provided the list of unmapped sequences, which were removed from the PyroTRF-ID pipeline.

### Generation of dT-RFLP profiles

Sequences that passed through all previous steps of the procedure were digested *in silico* using the restriction enzyme *Hae*III which was selected from the Bio.Restriction BioPython database. The dT-RFLP profiles were generated for each sample considering both the size of the dT-RFs and their relative abundance in the sample. Sequences containing no restriction site were discarded. A raw dT-RFLP profile plot was generated as output file. Different restriction enzymes can be tested in the PyroTRF-ID workflow for the optimization of dT-RFLP profiles. This is particularly convenient for designing new eT-RFLP approaches. Such screening can be performed on the pyrosequencing datasets without requirements of eT-RFLP data as input file.

### Comparison of eT-RFLP and dT-RFLP profiles

In order to allow comparison with eT-RFLP profiles, T-RFs below 50 bp were removed, and a second set of dT-RFLP profiles was generated. To overcome any possible discrepancy between experimental and *in silico* T-RFLP [30], PyroTRF-ID evaluated the most probable drift between e- and dT-RFLP profiles by computing the cross-correlation of the two. A plot showing the results of the cross-correlation was generated in order to help the user assessing the optimal shift to apply for aligning both profiles. By default, PyroTRF-ID corrected the dT-RFLP profile based on the drift with the highest cross-correlation. However, the user can optionally define a specific shift to apply. After shifting the dT-RFLP data, a mirror plot was generated allowing visual comparison of the dT-RFLP and eT-RFLP profiles.

### Assignment of affiliation to dT-RFs

Peak annotation files were generated in comma-separated-values format (.csv), listing all digitally obtained T-RFs within each dT-RFLP profile, together with their original and shifted lengths. Closest phylogenetic affiliations were provided together with the number of reads and their relative contribution to the T-RF, as well as with the absolute and normalized SW mapping scores, and the Genbank code of each reference sequence. When eT-RFLP data were not provided in the workflow, the peak annotation file was directly obtained after dT-RFLP processing without removing dT-RFs below 50 bp and without indication of T-RF shift.

### Optimization and testing of PyroTRF-ID

The initial testing and validation steps were carried out with the 17 pyrosequencing datasets originating from the two environments. The impact of the data processing steps of the PyroTRF-ID pipeline was assessed using two samples (GRW01 and AGS01). Three different combinations of algorithms were tested for the processing of sequences (Table 1), and their respective impact on the final dT-RFLP profiles was compared by calculating richness and Shannon′s H′ diversity indices. The aim was to optimize the cross-correlation between dT-RFLP and the corresponding eT-RFLP profiles. The optimal standardized PyroTRF-ID procedure was selected based on this assessment.

**Table 1:** Combinations of algorithms tested for the processing of pyrosequencing datasets for dT-RFLP profiling in PyroTRF-ID

**Pyrosequencing data processing procedure**	**Processing algorithms**				
	**PHRED-filtering**^**a**^	**Sequence length cut-off**	**Denoising**	**Filtering by SW mapping score**^**b**^	**Restriction of sequences**^**c**^
1) Standard dT-RFLP^d^	>20^e^	>300 bp	Yes	>150^f^	Yes
2) Filtered dT-RFLP^e^	>20	>300 bp	No	>150	Yes
3) Raw dT-RFLP^d^	>20	>300 bp	No	No (0)^g^	Yes

^a^ PHRED score = −10 log P_error_ with P_error_ = 10^-PHRED/10^ as the probability that a base was called incorrectly. For all trials, the raw pyrosequencing datasets were systematically filtered according to the PHRED quality score. Only sequences with a related PHRED score above 20 were conserved. This corresponds to a P_error_ of 1/100.

^b^ A SW mapping score of 150 was set as cutoff. In the case when sequences were preliminarily denoised, it was nevertheless observed that no denoised sequence was rejected at the mapping stage. Processing without filtering by the SW mapping score was done by setting a cutoff of 0.

^c^ The processed sequences were digested *in silico* with the restriction enzyme.

^d^ The first combination with denoising was defined as the standard PyroTRF-ID procedure.

^e^ In the second combination, only a filtering method at the mapping stage was considered.

^f^ In the third combination, raw datasets of sequences obtained after PHRED-filtering of the pyrosequencing datasets were digested without post-processing.

The optimal procedure was then applied for the comparison of PyroTRF-ID results obtained from groundwater and wastewater environments. Finally, restriction enzymes commonly used in T-RFLP analyses of bacterial communities (*Alu*I, *Hha*I, *Msp*I, *Rsa*I, *Taq*I, and *Hae*III) were selected for comparison of profiling resolutions. Visual observation, richness and diversity indices, as well as density plots were used to analyze the distributions of T-RFs along the e- and dT-RFLP profiles.

## Results

### Pyrosequencing quality control and read length limitation

The principal quality outputs given by PyroTRF-ID are presented in Additional file 1 for the low throughput (LowRA) and high throughput (HighRA) pyrosequencing methods used in this study. On average, 6′380 and 32′480 reads were obtained for each method, respectively.

Filtering based on the PHRED quality criterion allowed discarding low quality sequences. Most of the remaining sequences had a length below 400–450 bp (Additional file 1a). For the LowRA and HighRA methods, the median number of reads (800 and 2′750) was related to a PHRED score of 30 and 35, respectively, and more than 99% of reads were related to a PHRED score above 20 (Additional file 1b). Only reads longer than 300 bp were conserved for subsequent *in silico* digestion, because including short sequences in the dT-RFLP profiles may have altered the relative proportions of T-RFs to eT-RFLP profiles. Pyrosequencing datasets obtained with the HighRA method were predominantly composed of short reads below 300 bp (69% of a total of 24′810 reads in the example presented, Additional file 1c). However, 7′641 reads (31%) of high quality sequences were still available for PyroTRF-ID analysis, which was even larger than the number of high quality sequences remaining with the LowRA method (2′804 reads, 47%).

### Effect of denoising and mapping procedures

Denoising of pyrosequencing datasets was performed in order to correct for classical 454 analytical errors including the above-mentioned cut-off values: a minimum PHRED quality score of 20, as well as minimum and maximum sequence lengths of 300 and 500 bp, respectively. The denoising process generated a subset of representative sequences harboring at least 3% dissimilarity to each other. This amounted to 17±1% and 43±9% of the number of reads present in the raw datasets obtained with the HighRA and LowRA methods, respectively.

After denoising, the mapping process was the time-limiting step in the PyroTRF-ID pipeline. Twenty minutes were required for mapping the largest datasets against the Greengenes database. Discarding sequences shorter than 300 bp did not lead to a reduced number of detected bacterial phylotypes (Additional file 2). Bacterial community compositions obtained both without and with minimum sequence length cut-off exhibited high correspondences with determination coefficients of R^2^ between 0.80 and 0.99 depending on the sample type and the reference database used for mapping (Greengenes and RDP). Within the sets of identified phlyotypes, sequences affiliated to *Geobacter* sp. displayed the highest difference in relative abundance (18%), resulting from a high proportion of short reads below 200 bp in the dataset GRW01.

After PHRED-filtering, the remaining raw sequences had maximum lengths of 450 bp and therefore the maximal SW mapping scores amounted to around 450. The distributions of the absolute and normalized SW scores are provided in Additional file 3, and are compared to the distribution of the sequence identity score, usually used for phylogenetic affiliation of sequences. These two scoring methods are conceptually different, since nucleotide positions and gaps are taken into account in the computation of SW scores. The median absolute and normalized SW scores amounted to 270 and 0.736, respectively. The relative number of bacterial affiliations obtained with normalized SW scores higher than 0.600 and 0.900 amounted to 89% and 37%, respectively. A total of 81% of the affiliations up to the genus level were related to a sequence identity score of 100%, and 91% with an identity score above 97%. The normalized SW scores obtained for the predominant affiliations presented in Tables 2 and 3 were comprised between 0.630 and 1.000, and are most likely related to sequence identity scores above 97%.

**Table 2:** Phylogenetic annotation of identified T-RFs

**eTRF**^**a**^**(bp)**	**dTRF**^**a**^**(bp)**	**dTRF shifted**^**b**^**(bp)**	**Counts**^**c**^**(−)**	**Relative contribution to T-RF**^**d**^**(%)**	**Phylogenetic affiliation**^**e**^	**Reference OTU**^**f**^	**Reference GenBank accession number**^**g**^	**SW mapping score**^**h**^**(−)**	**Normalized SW mapping score**^**i**^**(−)**
**Aerobic granular sludge biofilms from wastewater treatment reactors**									
n.a. (32)^j^	39	34	550	70.6	F: *Xanthomonadaceae*	4015	GQ396926	386	0.960
			(276)	(35.0)	(G: *Thermomonas*)	(4045)	(EU834762)	(452)	(0.983)
			(128)	(16.0)	(G: *Pseudoxanthomonas*)	(4035)	(EU834761)	(385)	(0.955)
			112	14.3	O: *Flavobacteriales*	1151	AY468464	434	1.000
			46	5.9	F: *Rhodobacteraceae*	2718	AY212706	448	1.000
			37	4.8	S: *Rhodocyclus tenuis*	3160	AB200295	363	0.917
			18	2.3	O: *Sphingobacteriales*	1229	GU454872	394	0.990
			5	0.6	C: *Gammaproteobacteria*	3370	AY098896	403	0.906
			4	0.5	O: *Rhizobiales*	2549	EU429497	360	0.981
			4	0.5	O: *Myxococcales*	3246	DQ228369	302	0.765
			1	0.1	O: *Bacteroidales*	991	EU104248	180	0.636
194	198	193	10	90.9	G: *Acidovorax*	3011	AJ864847	384	1.000
			1	9.1	F: *Xanthomonadaceae*	4035	EF027004	303	0.819
214	219	214	769	99.6	S: *Rhodocyclus tenuis*	3160	AB200295	371	0.949
			1	0.1	G: *Methyloversatilis*	3158	DQ066958	368	0.958
			1	0.1	G: *Dechloromonas*	3156	DQ413103	321	0.988
			1	0.1	G: *Nitrosomonas*	3136	EU937892	278	0.753
220	225	220	50	92.6	O: *Rhizobiales*	2580	NR025302		
			(31)	(57.0)	(G: *Aminobacter*)				
			2	3.7	S: *Rhodocyclus tenuis*	3160	AB200295	206	0.703
			1	1.9	F: *Hyphomonadaceae*	2656	AF236001	229	0.636
			1	1.9	P: *Firmicutes*	2235	DQ413080	284	1.000
216	221	216	10	34.5	S: *Rhodocyclus tenuis*	3160	AF502230	296	0.773
			8	27.6	G: *Nitrosomonas*	3136	GU183579	364	0.948
			6	20.7	C: *Anaerolineae*	1317	EU104216	202	0.598
			3	10.3	G: *Methyloversatilis*	3158	CU922545	360	0.909
			1	3.4	G: *Aminobacter*	2580	L20802	281	0.829
			1	3.4	G: *Dechloromonas*	3156	DQ413103	273	0.898
223	228	223	44		F: *Intrasporangiaceae*	418	AF255629		
					(G: *Tetrasphaera*)				
			15	24.6	F: *Hyphomonadaceae*	2656	AF236001	298	0.674
			1	1.6	F: *Microbacteriaceae*	441	GQ009478	228	0.544
			1	1.6	O: *Acidimicrobiales*	268	GQ009478	153	0.447
239	243	238	275	98.9	C: *Gammaproteobacteria*	3370	EU529737	446	0.982
			2	0.7	G: *Leptospira*	4092	AB476706	350	0.926
			1	0.4	P: *Armatimonadetes*	975	EU332819	275	0.846
249	253	249	9	100.0	S: *Rhodocyclus tenuis*	3160	AB200295	228	0.752
255	258	253	7	100.0	O: *Sphingobacteriales*	1171	FJ793188	355	0.989
260	263	258	16	94.1	G: *Nitrospira*	2360	GQ487996	389	0.982
			1	5.9	O: *Sphingobacteriales*	1171	FJ536916	251	0.640
260	264	259	38	97.4	O: *Sphingobacteriales*	1170	EU104185	267	0.706
			1	2.6	G: *Nitrospira*	2360	GQ487996	319	0.788
297	302	297	26	100.0	G: *Herpetosiphon*	1359	NC009972	339	0.867
307	311	306	38	97.4	P: *Armatimonadetes*	975	CU921283	218	0.472
			1	2.6	O: *Sphingobacteriales*	1171	EU104210	196	0.525
321	323	318	17	100.0	G: *Cytophaga*	1208	EU104191	367	0.968
393	397	392	33	100.0	G: *Bdellovibrio*	3173	CU466777	262	0.663
**Groundwater samples from chloroethene-contaminated aquifers**									
63	69	64	93	85.3	F: *Methylococcaceae*	3686	AB354618	432	0.915
			14	12.8	F: *Crenotrichaceae*	3681	GU454947	290	0.816
			1	0.9	F: *Ectothiorhodospiraceae*	3510	AM902494	168	0.542
			1	0.9	P: candidate phylum OP3	2388	GQ356152	187	0.488
165	168	163	143	100.0	G: *Dehalococcoides*	1368	EF059529	448	0.953
190	193	191	12	54.6	F: *Desulfobulbaceae*	3177	AJ389624	379	0.945
			4	13.6	F: *Sphingomonadaceae*	2880	AY785128	263	0.555
			2	9.1	F: *Erythrobacteraceae*	2872	DQ811848	343	0.771
			2	9.1	C: *Alphaproteobacteria*	2451	AY921822	337	0.926
			1	4.6	F: *Rhodospirillaceae*	2793	AY625147	294	0.679
			1	4.6	F: *Rhodobiaceae*	2641	AB374390	328	0.877
198	201	196	140	98.6	G: *Desulfovibrio*	3215	FJ810587	473	1.000
			2	1.4	F: *Comamonadaceae*	3039	FN428768	311	0.814
210	214	209	233	98.3	F: *Dehalococcoidaceae*	1367	EU679418	262	0.665
			2	0.8	O: *Burkhorderiales*	3009	AM777991	367	0.927
			1	0.4	F: *Spirochaetaceae*	4130	EU073764	295	0.848
			1	0.4	P: candidate phylum TM7	4379	DQ404736	277	0.723
216	221	216	1010	90.9	F: *Gallionellaceae*	3080	EU802012	353	0.869
			94	8.5	G: *Rhodoferax*	3050	DQ628925	369	0.920
			3	0.3	G: *Methylotenera*	3093	AY212692	291	0.744
			1	0.1	G: *Methyloversatilis*	3158	GQ340363	296	0.765
			1	0.1	F: *Clostridiaceae*	2005	AJ863357	338	0.833
			1	0.1	C: *Anaerolineae*	1315	AB179693	229	0.511
			1	0.1	C: *Actinobacteria*	949	EU644115	372	0.907
243	247	243	389	99.7	F: *Dehalococcoidaceae*	1367	EU679418	255	0.631
			1	0.3	F: *Anaerolinaceae*	1321	AB447642	253	0.806

^a^ Experimental (eT-RF) and digital T-RFs (dT-RF).

^b^ Digital T-RF obtained after having shifted the digital dataset with the most probable average cross-correlation lag.

^c^ Number of reads of the target phylotype that contribute to the T-RF.

^d^ Diverse bacterial affiliates can contribute to the same T-RF.

^e^ Phylogenetic affiliation of the T-RF (K: kingdom, P: phylum, C: class, O: order, F: family, G: genus, S: species). Only the last identified phylogenetic branch is presented here.

^f^ Reference operational taxonomic unit (OTU) from the Greengenes public database related with the best SW mapping score. In the Greengenes taxonomy, OTU refer to terminal levels at which sequences are classified.

^g^ GenBank accession numbers provided by Greengenes for reference sequences.

^h^ Best SW mapping score obtained. SW scores consider nucleotide positions and gaps. The highest SW mapping score that can be obtained for a read is the length of the read itself.

^i^ SW mapping score normalized by the read length, as an estimation of the percentage of identity.

^j^ After having observed the presence of the dT-RF 34 bp, we returned to the raw eT-RFLP data and found an important eT-RF at 32 bp. However, Rossi et al. [8] considered that T-RFs below 50 bp are inconsistent and lacks of precision in sizing. This peak was therefore initially not taken into account in the original eT-RFLP profiles.

**Table 3:** T-RF diversity for single phylogenetic descriptions

**Phylogenetic affiliation**	**dTRF (bp)**	**dTRF shifted**^**a**^**(bp)**	**Counts**^**b**^**(−)**	**Relative contribution to T-RF**^**c**^**(%)**	**Reference OTU**^**d**^	**Reference GenBank accession number**^**e**^	**SW mapping score**^**f**^**(−)**	**Normalized SW mapping score**^**g**^**(−)**
**Flocculent and aerobic granular sludge samples from wastewater treatment systems**								
*Rhodocyclus tenuis*	39	34	37	4.8	3160	AB200295	363	0.917
	199	194	1	25.0	3160	AB200295	248	0.648
	205	200	3	100.0	3160	AF204247	314	0.858
	210	205	1	100.0	3160	AF204247	211	0.699
	218	213	11	91.7	3160	AB200295	356	0.942
	**219**	**214**	**769**	**99.6**	**3160**	**AB200295**	**371**	**0.949**
	220	215	6	37.5	3160	AF502230	318	0.817
	221	216	1	7.7	3160	AF502230	276	0.865
	225	220	2	3.7	3160	AB200295	206	0.703
	252	247	3	100.0	3160	AB200295	305	0.762
	253	248	9	100.0	3160	AB200295	228	0.752
	257	252	1	20.0	3160	AF502230	241	0.660
**Groundwater samples from aquifers contaminated with chloroethenes**								
*Dehalococcoides* spp.	166	161	1	100.0	1368	EF059529	290	0.775
	**168**	**163**	**143**	**100.0**	**1368**	**EF059529**	**241**	**0.717**
	169	164	2	100.0	1368	EF059529	331	0.768
	170	165	2	100.0	1368	EF059529	241	0.693
	171	166	1	50.0	1368	EF059529	303	0.783
	173	168	1	100.0	1368	EF059529	241	0.717
	176	171	1	100.0	1369	DQ833317	211	0.687
	179	174	1	100.0	1369	DQ833317	193	0.629
	188	183	4	66.7	1369	DQ833340	464	0.947

^a^ Digital T-RF obtained after having shifted the digital dataset with the most probable average cross-correlation lag.

^b^ Number of reads of the target phylotype that contribute to the T-RF.

^c^ Diverse bacterial affiliates can contribute to the same T-RF.

^d^ Reference OTU from the Greengenes public database obtained after mapping.

^e^ GenBank accession numbers provided by Greengenes for reference sequences.

^f^ Best SW mapping score obtained.

^g^ SW mapping score normalized by the read length.

### Generation of digital T-RFLP profiles

The dT-RFLP profiles were successfully generated with the standard PyroTRF-ID procedure (Table 1) from denoised bacterial pyrosequencing datasets of the GRW and the AGS sample series (Additional file 4). With *Hae*III, 165±29 and 87±11 T-RFs were present in the dT-RFLP profiles of the GRW and AGS series, respectively. For all samples, only a reduced number of dT-RFs above 400 bp were obtained because of the low pyrosequencing quality at sequence lengths between 400 and 500 bp.

**Figure 2:**
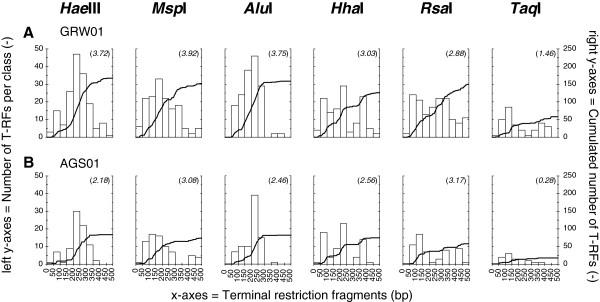
**Density plots displaying the repartition of T-RFs along the 0–500 bp domain with different endonucleases.** The effect of the different restriction endonucleases *Hae*III, *Alu*I, *Msp*I, *Hha*I, *Rsa*I and *Taq*I was tested on pyrosequencing datasets collected from the samples GRW01 (**A**) and AGS01 (**B**). Histograms represent the number of T-RFs produced per class of 50 bp (to read on the left y-axes). Thick black lines represent the cumulated number of T-RFs over the 500-bp fingerprints (to read on the right y-axes). The total cumulated number of T-RFs corresponds to the richness index. The number given in brackets corresponds to the Shannon′s diversity index.

An additional feature of PyroTRF-ID is the generation of dT-RFLP profiles with any restriction enzyme. Here profiles were obtained with five additional restriction enzymes and compared. Profiles of GRW samples were on average 2.3 times richer than ones of AGS samples, and each restriction enzyme generated characteristic dT-RFLP features regardless of the sample complexity (Figure 2 and Additional file 4). *Hae*III provided dT-RFLP profiles with the highest richness. The use of this enzyme resulted in the generation of dT-RFs stacked mainly between 200 and 300 bp. Highest diversities in dT-RFLP profiles were obtained with *Msp*I and *Rsa*I, respectively. Digestion with *Msp*I resulted in the most homogeneous distributions of dT-RFs up to approximately 300 bp. With the exception of *Hha*I, endonucleases did not produce numerous dT-RFs in the second half of the profiles, and cumulative curves flattened off. With *Hha*I, the cumulative curves increased step-wise. *Rsa*I resulted in dT-RFLP profiles displaying homogeneous distributions of dT-RFs for GRW samples, but lower diversity than *Hae*III, *Alu*I, *Msp*I, and *Hha*I. *Taq*I always provided profiles with the lowest richness and diversity.

### Comparison of digital and experimental T-RFLP profiles

**Figure 3:**
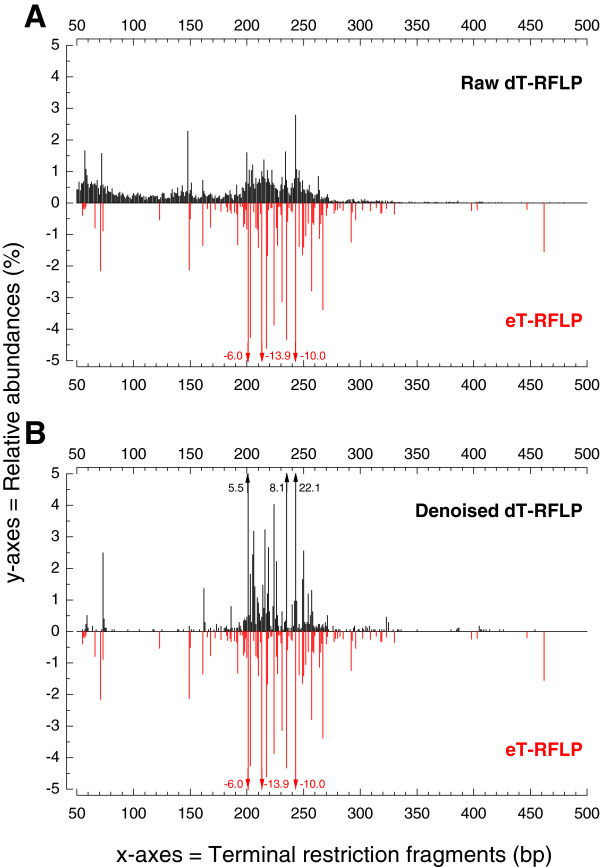
**Mirror plot displaying the cross-correlation between digital and experimental T-RFLP profiles.** This mirror plot was generated for the complex bacterial community of sample GRW01. Comparison of mirror plots constructed with raw (**A**) and denoised sequences (**B**). Relative abundances are displayed up to 5% absolute values. For those T-RFs exceeding these limits, the actual relative abundance is displayed beside the peak.

Mirror plots generated by PyroTRF-ID computed with raw and denoised pyrosequencing datasets obtained from a complex bacterial community (GRW01) are presented in Figure 3. Further examples of mirror plots are available in Additional file 5. Digital profiles generated from raw pyrosequencing datasets displayed Gaussian distributions around the most dominant dT-RFs of neighbor peaks (Figure 3a) which exhibited identical bacterial affiliations (data not shown). This feature was attributed to errors of the 454 pyrosequencing analysis. Denoised dT-RFLP profiles displayed enhanced relative abundances of dominant peaks and had a higher cross-correlation with eT-RFLP profiles (Figure 3b). By selecting representative sequences (so-called centroids) for clusters containing reads sharing at least 97% identity, in the QIIME denoising process, all neighbor peaks were integrated in the dominant dT-RFs resulting from the centroid sequences. Cross-correlations between dT-RFLP and eT-RFLP profiles issued from sample GRW01 increased from 0.43 to 0.62 after denoising of the pyrosequencing data.

The dT-RFLP profiles exhibited a drift of 4 to 6 bp compared to eT-RFLP profiles. From *in silico* restriction of a 150 clone library obtained in our laboratory using the TRiFLe software [30], we confirmed that *in silico* T-RFs were, on average, 4±1 bp (min 3 bp – max 6 bp) longer than the experimental ones (data not shown). After correcting with an optimal shift (Additional file 6), maximum cross-correlation coefficients between denoised dT-RFLP and eT-RFLP profiles ranged from 0.55±0.14 and 0.67±0.05 for the GRW samples (HighRA and LowRA method, respectively) to 0.82±0.10 for the AGS samples (LowRA method) (Table 4).

**Table 4:** Cross-correlations between experimental and standard digital T-RFLP profiles

**Samples**	**Optimal cross-correlation lag between digital and experimental T-RFLP profiles**^**a**^**(bp)**	**Maximum cross-correlation coefficient at optimal lag**^**b**^**(−)**	**Total number of experimental T-RFs per profile (−)**	**Number of experimental T-RFs affiliated with digital T-RFs**^**c**^**(−)**	**Percentage of experimental T-RFs affiliated with digital T-RFs**^**c**^**(%)**
**Groundwater**					
GRW01^d^	−4	0.62	88	58	66
GRW02^d^	−5	0.69	50	23	46
GRW03^d^	−4	0.44	76	62	82
GRW04^d^	−5	0.71	44	24	44
GRW05^d^	−5	0.35	75	56	75
GRW06^d^	−6	0.51	87	70	81
*Avg±stdev (min-max)*	*−5±1*	*0.55±0.14*	*70±19*	*49±20*	*67±14*
	*-(4–6)*	*(0.35-0.71)*	*(44–88)*	*(23–70)*	*(44–82)*
GRW07^e^	−6	0.70	57	17	30
GRW08^e^	−4	0.59	54	43	80
GRW09^e^	−4	0.69	71	66	93
GRW10^e^	−5	0.68	70	22	31
*Avg±stdev (min-max)*	*−5±1*	*0.67±0.05*	*59±11*	*34±20*	*59±33*
	*-(4–6)*	*(0.59-0.70)*	*(44–71)*	*(17–66)*	*(30–93)*
**Aerobic granular sludge**					
AGS01^e^	−5	0.75	48	31	65
AGS02^e,f^	−5	0.90	38	22	58
AGS03^e,f^	−5	0.90	38	19	50
AGS04^e^	−5	0.72	52	24	46
AGS05^e^	−4	0.67	43	29	67
AGS06^e,f^	−5	0.91	38	19	50
AGS07^e^	−5	0.80	38	31	82
*Avg±stdev (min-max)*	*−5±0*	*0.82±0.10*	*42±6*	*25±5*	*61±12*
	*-(4–5)*	*(0.67-0.91)*	*(38–52)*	*(19–31)*	*(46–82)*

^a^ Shift leading to optimal matching of the digital to the experimental T-RFLP profile.

^b^ Maximum cross-correlation coefficients obtained after matching of the digital to the experimental T-RFLP profile.

^c^ Number and percentage of experimental T-RFs having corresponding digital T-RFs.

^d^ Samples GRW01-06 were pyrosequenced with the HighRA method.

^e^ Samples GRW07-10 and AGS01-07 were pyrosequenced with the LowRA method.

^f^ Samples AGS02, AGS03, and AGS06 are triplicates from the same DNA extract.

### Impact of sequence processing steps, pyrosequencing methods and sample types

**Figure 4:**
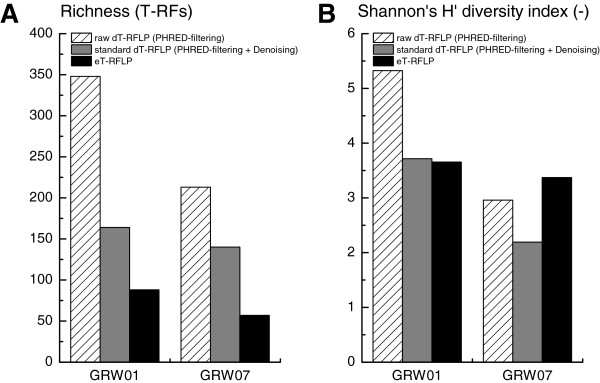
**Assessment of the impact of data processing on dT-RFLP profiles, and comparison with eT-RFLP profiles.** Richness and Shannon′s H′ diversity indices were calculated in a way to quantify the impact of the pyrosequencing data processing parameters on the resulting dT-RFLP profiles. Two examples are given for samples pyrosequenced with the HighRA (GRW01) and LowRA methods (GRW07).

Indices of richness (number of T-RFs) and diversity (number of T-RFs and distributions of abundances) were used to evaluate the impacts of data processing steps, pyrosequencing methods and sample types on the structure of the final dT-RFLP profiles (Figure 4). The changes of the indices were considered positive if they approached the indices determined for eT-RFLP profiles. The raw dT-RFLP profiles were composed of 2.4- to 7.4-times more T-RFs than the eT-RFLP profiles. Denoising resulted in a decrease of richness and diversity. The ratios of richness and diversity between standard dT-RFLP and eT-RFLP profiles amounted to 2.5±0.6 and 1.0±0.3, respectively, for high-complexity samples (GRW), and to 2.1±0.5 and 0.8±0.2, respectively, for low-complexity samples (AGS). The raw dT-RFLP profiles of the groundwater samples GRW01-GRW06, which were sequenced with the HighRA method, were composed of 4 to 7.4-times more T-RFs than their respective eT-RFLP profiles. Groundwater samples GRW07-GRW10 sequenced with the LowRA method displayed ratios of raw dT-RFs to eT-RFs which were between 2.4 and 5.2. After denoising, both sets of groundwater-related dT-RFLP profiles exhibited similar richness and diversity and were closer to indices of eT-RFLP profiles than raw dT-RFLP profiles (Figure 4).

The DNA extract of one AGS sample was analyzed in triplicate from pyrosequencing to PyroTRF-ID. The resulting standard dT-RFLP profiles contained 94±10 T-RFs, and exhibited very close diversity indices of 1.48±0.03. In comparison, denoised profiles of all AGS samples collected over 50 days contained similar numbers of T-RFs (84±9) but exhibited quite different diversity indices of 2.12±0.48. There was also very little variation in the cross-correlation coefficients (0.90±0.01) between the dT-RFLP profiles and the corresponding eT-RFLP profile. All three denoised T-RFLP profiles exhibited similar structures, and affiliations were the same for T-RFs that could be identified.

### Efficiency of phylogenetic affiliation of T-RFs

**Figure 5:**
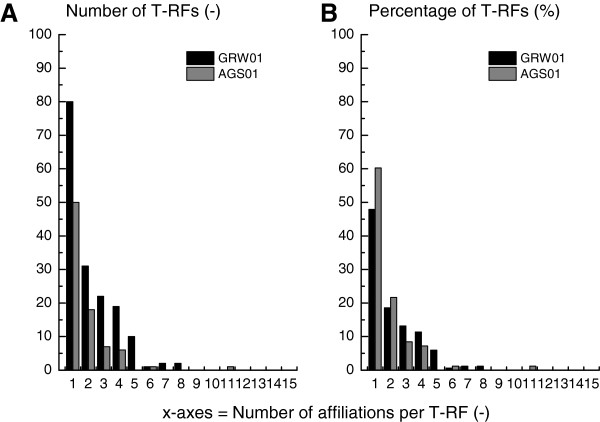
**Amount of bacterial affiliations contributing to T-RFs.** The absolute (**A**) and relative numbers (**B**) of T-RFs that comprised one to several bacterial affiliations is given for the samples GRW01 and AGS01.

Comprehensive phylogenetic information was provided by PyroTRF-ID for each dT-RF, as exemplified in Table 2. Depending on the sample type, between 45 and 60% of all dT-RFs were affiliated with a unique bacterial phylotype (Figure 5). The other dT-RFs were affiliated with two or more phylotypes, showing different contribution patterns. In such cases, a single phylotype was usually clearly predominating with a relative contribution ranging from 50 to 99%. However, for some T-RFs no clear dominant phylotype emerged (e.g. eT-RF 216 in AGS samples, Table 2).

Some reference sequences were sometimes represented by several T-RFs (Table 3). For instance, in AGS01, six dT-RFs (34, 194, 213, 214, 220, 247 bp) were affiliated to the same reference sequence of *Rhodocyclus tenuis* (accession number AB200295), with shifted T-RF 214 being predominant (769 of 844 reads). The *Dehalococcoides* sp. affiliation in sample GRW05 was related to eight T-RFs, with shifted T-RF 163 being predominant (143 of 156 reads). To investigate this phenomenon, reads resulting in different *Dehalococcoides*-affiliated T-RFs were retrieved from the pyrosequencing dataset and aligned with ClustalX (Additional file 7). This analysis showed that the multiple T-RF sizes observed were due to reads harboring insertions or deletions of nucleotides before the first *Hae*III restriction site or to nucleotide modifications within *Hae*III sites.

## Discussion

### Advantages and novelties of the PyroTRF-ID bioinformatics methodology

This study describes the development of the PyroTRF-ID bioinformatics methodology for the analysis of microbial community structures, and its application on low- and high-complexity environments. PyroTRF-ID can be seen as the core of a high-throughput methodology for assessing microbial community structures and their dynamics combining NGS technologies and more traditional community fingerprinting techniques such as T-RFLP. More than just predicting the most probable T-RF size of target phylotypes, PyroTRF-ID allows the generation of dT-RFLP profiles from 16S rRNA gene pyrosequencing datasets and the identification of experimental T-RFs by comparing dT-RFLP to eT-RFLP profiles constructed from the same DNA samples.

At the initial stage of the assessment of a microbial community, PyroTRF-ID can be used for the design of an eT-RFLP procedure adapted to a given microbial community through digital screening of restriction enzymes. In contrast to previous studies involving *in silico* restriction of artificial microbial communities compiled from selected reference sequences from public or cloning-sequencing databases [25,29,31], PyroTRF-ID works on sample-based pyrosequencing datasets. This requires the pyrosequencing of a limited number of initial samples. The number of T-RFs, the homogeneity in their distribution, and the number of phylotypes contributing to T-RFs should be used as criteria for the choice of the best suited enzyme. Combination of pyrosequencing and eT-RFLP datasets obtained on the same initial set of samples enables the beginning of the study of new microbial systems with knowledge on T-RFs affiliation. The length of T-RFs and their sequences are directly representative of the investigated sample rather than inferred from existing databases. In this sense, the complexity of the original environment is accurately investigated. For all types of low- and high-complexity environments assessed in this study, *Hae*III, *Alu*I and *Msp*I were good candidates for the generation of rich and diverse dT-RFLP profiles.

Subsequently, eT-RFLP can be used as a routine method to assess the dynamics of the stuctures of microbial communites, avoiding the need for systematic pyrosequencing analyses. We suggest that pyrosequencing should be applied at selected time intervals or on representative samples to ensure that the T-RFs still display the same phylogenetic composition. Combining T-RFLP and pyrosequencing is particularly adapted for the temporal follow-up of a microbial system, taking advantage of the relative low costs of T-RFLP and its convenience for routine assessment of microbial community structures, and of the power of pyrosequencing for assessing the composition of these communities. PyroTRF-ID has already been used for the study of bacterial communities involved in start-up of aerobic granular sludge systems [34] and in natural attenuation of chloroethene-contaminated aquifers [33].

### Performance assessment and limitations of PyroTRF-ID

Classical 454 pyrosequencing errors, such as, inaccurate resolving of homopolymers and single base insertions [54], were expected to impact the quality of dT-RFLP profiles by overestimating the number of dT-RFs present [55,56]. The use of a denoising procedure based on the analysis of rank-abundance distributions [47] was a prerequisite to minimize pyrosequencing errors and to generate dT-RFLP profiles approaching the structure of eT-RFLP profiles, as assessed by the improved cross-correlation coefficients. Filtering pyrosequencing reads with the SW mapping score threshold only slightly reduced overestimations. In addition, this filtering approach does not specifically remove reads based on their intrinsic quality but rather on similarities with existing sequences from the database, hence reducing the complexity of the studied bacterial community to what is already known [54,57]. When denoising was applied, the use of a SW mapping score threshold did not improve the shape of dT-RFLP profiles. Whereas small-size reads were more abundant in the HighRA pyrosequencing datasets. The pyrosequencing method and the initial amount of reads did not impact the final PyroTRF-ID output. Only the level of complexity of the bacterial communities of the ecosystems could have explained the differences in richness among T-RFLP profiles.

Clipping the low-quality end parts of sequences is an option to improve sequence quality but it is quite improbable that it has an impact on the outcome of the taxon assignment and the creation of dT-RFLP profile. When PyroTRF-ID is run with the “--qiime” option, quality trimming is done using the protocol proposed in QIIME [43] and its online tutorial (http://qiime.org/tutorials/denoising_454_data.html). This includes the amplicon noise procedure that is efficient in correcting for sequencing errors, PCR single base substitutions, and PCR chimeras [58]. Even if some wrong base calls remain in the consensus sequences after this, they should not affect the assignment to taxon as the BWA aligner can account for mismatches. It should not influence the dT-RFLP profile either since a mismatch outside of the enzyme cleavage site does not affect the length of the fragment produced. As the fragment length is determined by counting the number of base pairs before the enzyme cleavage site and that the BWA aligner does not necessarily use the whole sequence when selecting a match, clipping the low-quality ends of sequences would probably have no measurable effect.

Discrepancies of 0–7 bp between the size of *in silico* predicted T-RFs and eT-RFs have previously been reported [30,59]. In the present study, an average discrepancy of 4–6 bp was observed between dT-RFLP and eT-RFLP profiles. This drift was confirmed by comparison of *in silico* and experimental digestion of 150 clones from a clone library. To overcome the bias induced by the experimental drift, we introduced the calculation of a cross-correlation between dT-RFLP and eT-RFLP profiles. The entire dT-RFLP profile was shifted by the number of base pairs enabling better fitting to the corresponding eT-RFLP profile. It is known that the drift is not constant across the T-RFs but rather depends on the true T-RF length, on its purine content, and on its secondary structure [59-61]. Mirror plots sometimes displayed a 1-bp difference between eT-RFs and dT-RFs. It was crucial for the user to visually inspect the mirror plots prior to semi-manually assigning phylotypes to eT-RFs. The approach adopted here consisted of selecting eT-RFs to identify prior to checking their alignment with dT-RFs. In order to overcome manual inspection, a shift could be computed for each single dT-RF in relation with its sequence composition and theoretical secondary structure [60]. However, the standard deviation associated with this method is still higher than 1 bp. Shifting each single dT-RF based on this function was therefore not expected to improve the alignment accuracy. If at a later stage an improved method for calculating drift for single dT-RFs will be available, it could replace our approach combining a shift of the whole profile, cross-correlation calculation between dT-RFLP and eT-RFLP profiles, and manual inspection. Though user interpretation can introduce a subjective step, final manual processing of T-RFLP profiles can remain the only way to resolve T-RF alignment problems [59]. We nevertheless suggest that selected samples of the investigated system should pass through PyroTRF-ID in triplicates in order to validate the optimal drift determined in the cross-correlation analysis.

Following the standard PyroTRF-ID procedure, high level of correspondence was obtained between dT-RFLP and eT-RFLP profiles. Over all samples, 63±18% of all eT-RFs could be affiliated with a corresponding dT-RF. Correspondence between dT-RFs and eT-RFs was relatively obvious for high abundance T-RFs, in contrast to low abundance dT-RFs. Numerous low abundance dT-RFs were present in dT-RFLP profiles but absent in eT-RFLP profiles. Conversely, eT-RFs were sometimes lacking a corresponding dT-RF. This mainly occurred in profiles generated using pyrosequencing datasets with an initially low amount of reads exceeding 400 bp. The lower proportion of long reads was associated with a decreasing probability of finding a restriction site in the final portion of the sequences. For eT-RFs near 500 bp, incomplete enzymatic restriction could explain that undigested amplicons were detected in the electrophoresis runs [62,63]. These features, however, do not explain missing dT-RFs, which sometimes occurred in the initial portion of the dT-RFLP profile. Egert and Friedrich [64] have attributed the presence of ′pseudo T-RFs′ to undigested single stranded DNA amplicons, and have cleared them by cleaving amplicons with single-strand-specific mung bean nuclease. An interesting possibility to increase considerably the number of long reads would be to use bidirectional reads as used by Pilloni et al. for the characterization of tar-oil-degrading microbial communities [65].

The majority of dT-RFs were affiliated to several phylotypes, revealing the underlying phylogenetic complexity, which was in agreement with Kitts [59]. PyroTRF-ID enabled assessing the relative contributions of each phylotype, and determining the most abundant ones. In most cases, one phylotype clearly displayed the highest number of reads for one dT-RF. However, for some dT-RFs several phylotypes contributed almost equally to the total number of reads. Although problematic while aiming at identifying T-RFs, this information is of primary importance if PyroTRF-ID is intended to be used for designing the most adapted T-RFLP procedure for the study of a particular bacterial community. Finally, as exemplified by Additional file 2, the reference mapping database can have an impact on the identification of T-RFs. A fraction of 35 to 45% of the reads was unassigned during mapping in MG-RAST with the Greengenes database, while only 3-5% was unassigned with RDP. This aspect stresses the need of standardized databases and microbiome dataset processing approaches in the microbial ecology field.

## Conclusions

This study presented the successful development of the PyroTRF-ID bioinformatics methodology for high-throughput generation of digital T-RFLP profiles from massive sequencing datasets and for assigning phylotypes to eT-RFs based on pyrosequences obtained from the same samples. In addition, this study leads to the following conclusions:

• The combination of pyrosequencing and eT-RFLP data directly obtained from the same samples was a powerful characteristic of the PyroTRF-ID methodology, enabling generation of dT-RFLP profiles that integrate the whole complexity of microbiomes of interest.

• The LowRA and HighRA 454 pyrosequencing method did not impact on the final results of the PyroTRF-ID procedure.

• As in any new generation sequencing analysis, denoising was a crucial step in the 454 pyrosequencing dataset processing pipeline in order to generate representative digital fingerprints.

• The PyroTRF-ID workflow could be applied to the screening of restriction enzymes for the optimization of favorably distributed eT-RFLP profiles by considering the entire underlying microbial communities. *Hae*III, *Msp*I and *Alu*I were good candidates for T-RFLP profiling with high richness and diversity indices.

• The PyroTRF-ID methodology was validated with different samples from low- and high-complexity environments, and could be implemented in a broad spectrum of biological samples in environmental to medical applications with optimized laboratory and computational costs. This methodology is probably not restricted to pyrosequencing datasets, and could be, after some modifications, applied to datasets obtained with any kind of sequencing techniques.

## Competing interests

The authors declare that they have no competing interests.

## Authors’ contributions

DGW and NS equally contributed to this work by conceiving, designing and coordinating the study, by carrying out sampling and molecular biology investigations, by leading the development of the PyroTRF-ID bioinformatics methodology, by analyzing all collected data, and by drafting the manuscript. DGW additionally conceived the tables and the figures. LS was responsible for the optimization and validation of PyroTRF-ID and wrote the underlying codes. GL coded the initial bioinformatics procedure. JM and PR participated in the design of the study. JR coordinated the development of PyroTRF-ID at the Bioinformatics and Biostatistics Core Facility. CH led the project and gave the initial idea of reconstructing T-RFLP profiles from pyrosequencing data. DGW and NS wrote the manuscript, with additional contributions of JM, PR, and CH. All authors read and approved the final manuscript.

## Supplementary Material

Additional file 1 **Quality plots generated for samples pyrosequenced with LowRA (>3′000 reads) and HighRA methods (>10′000 reads).** Sequence quality PHRED scores over all bases **(A)**: PHRED scores are defined as the logarithm of the base-calling error probability P_error_ = 10^-PHRED/10^ and PHRED = −10 log P_error_. Box plots represent the distribution of reads quality at each sequence length. The black curve represents the mean sequence quality in function of the sequence length. Distribution of the mean sequence quality PHRED score over the pyrosequencing reads **(B)**. Distribution of sequence lengths over all pyrosequencing reads **(C)**. Only sequences between 300 and 500 bp were kept for dT-RFLP analysis. Click here for file

Additional file 2 **Assessment of mapping performances with pyrosequencing datasets denoised without (0–500 bp) and with (300–500 bp) minimal read length cutoff.** Examples are given for the groundwater sample GRW01, the flocculent activated sludge sample FLS01 and the aerobic granular sludge sample AGS01. After denoising with the one or the other method, each dataset was mapped against a reference database with MG-RAST [66]. No cutoff was set for e-value, minimum identity and minimum alignment length. After having observed that between 35-45% of the sequences were unassigned with Greengenes, RDP – the Ribosomal Database Project [67] was used as reference database for this assessment (only 4% unassigned sequences). Correlations between bacterial community profiles obtained with both denoising methods and both reference databases were analyzed with STAMP [68]. Click here for file

Additional file 3 **Comparison of the distributions of the SW mapping score and of the traditional identity score used by microbial ecologists in the field of environmental sciences for phylogenetic affiliation of sequences.** The distributions of the absolute SW score **(A)** and of the SW scores normalized by the read lengths **(B)** obtained after mapping of 15 pyrosequencing datasets with the BWA-SW algorithm implemented in the PyroTRF-ID methodology are compared to the distribution of the identity score obtained after annotation of 10 pyrosequencing datasets with MG-RAST [66]**(C)**. Greengenes was used as annotation source in all cases. The obtained distributions are characterized by median (m), average (avg) and standard deviation values (s). Click here for file

Additional file 4 **Full digital T-RFLP profiles.** Examples of full digital T-RFLP profiles obtained with the restriction enzymes *Hae*III and *Msp*I for the samples GRW01 **(A)** and AGS01 **(B)**. Click here for file

Additional file 5 **Comparison of mirror plots obtained on raw** (left) **and on denoised** (right) **pyrosequencing datasets.** Examples are given for the sample GRW01 pyrosequenced with the HighRA method **(A)** and for the samples GRW07 **(B)** and AGS01 **(C)** pyrosequenced with the LowRA method. Click here for file

Additional file 6 **Assessment of cross-correlation and optimal lag between denoised dT-RFLP and eT-RFLP profiles.** The denoised dT-RFLP profiles of the samples AGS07 **(A)** and GRW04 **(B)** were both shifted with optimal lags of −5 bp to match with the related eT-RFLP profiles. At these optimal lags, the maximum cross-correlation coefficients amounted to 0.91 (AGS07) and 0.71 (GRW04). Click here for file

Additional file 7 **Alignment of sequences mapping with the same reference sequence with identical accession number in the Greengenes database, and resulting in different digital T-RFs.** Examples are given for the *Rhodocyclus tenuis* affiliates (accession number AB200295) of sample AGS01 and for *Dehalococcoides* relatives (accession number EF059529) of sample GRW05. Click here for file
