# Supplementary material for: PyroTRF-ID: a novel bioinformatics methodology for the affiliation of terminal-restriction fragments using 16S rRNA gene pyrosequencing data
Source: BMC Microbiol. 2012 Dec 27;12:306. doi: 10.1186/1471-2180-12-306 (PMC3566925; doi:10.1186/1471-2180-12-306)
Supplement: Additional file 1 — Quality plots generated for samples pyrosequenced with LowRA (>3′000 reads) and HighRA methods (>10′000 reads). Sequence quality PHRED scores over all bases (A): PHRED scores are defined as the logarithm of the base-calling error probability Perror = 10-PHRED/10 and PHRED = −10 log Perror. Box plots represent the distribution of reads quality at each sequence length. The black curve represents the mean sequence quality in function of the sequence length. Distribution of the mean sequence quality PHRED score over the pyrosequencing reads (B). Distribution of sequence lengths over all pyrosequencing reads (C). Only sequences between 300 and 500 bp were kept for dT-RFLP analysis. [file 1471-2180-12-306-S1.pdf]

Weissbrodt D.G. and Shani N., et al., **Additional files**, PyroTRF-ID.

Additional file 1

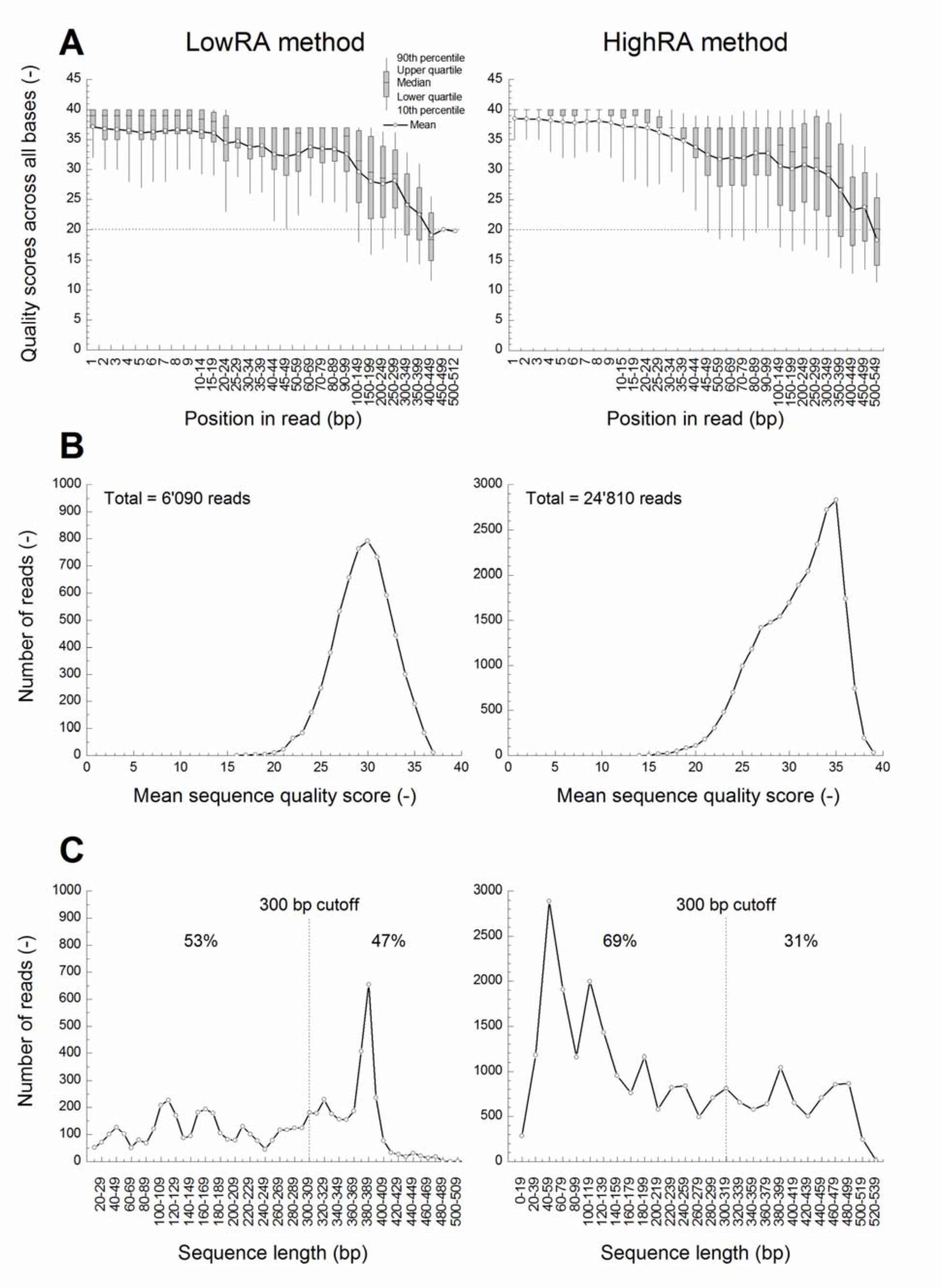

Figure AF1.1 – Quality plots generated for samples pyrosequenced with LowRA (>3’000 reads) and HighRA methods (>10’000 reads).

Sequence quality PHRED scores over all bases **(A)**: PHRED scores are defined as the logarithm of the base-calling error probability $P_{error} = 10^{-PHRED/10}$ and PHRED = -10 log P_{error}. Box plots represent the distribution of reads quality at each sequence length. The black curve represents the mean sequence quality in function of the sequence length. Distribution of the mean sequence quality PHRED score over the pyrosequencing reads **(B)**. Distribution of sequence lengths over all pyrosequencing reads **(C)**. Only sequences between 300 and 500 bp were kept for dT-RFLP analysis.
