# Supplementary material for: PyroTRF-ID: a novel bioinformatics methodology for the affiliation of terminal-restriction fragments using 16S rRNA gene pyrosequencing data
Source: BMC Microbiol. 2012 Dec 27;12:306. doi: 10.1186/1471-2180-12-306 (PMC3566925; doi:10.1186/1471-2180-12-306)
Supplement: Additional file 2 — Assessment of mapping performances with pyrosequencing datasets denoised without (0–500 bp) and with (300–500 bp) minimal read length cutoff. Examples are given for the groundwater sample GRW01, the flocculent activated sludge sample FLS01 and the aerobic granular sludge sample AGS01. After denoising with the one or the other method, each dataset was mapped against a reference database with MG-RAST [66]. No cutoff was set for e-value, minimum identity and minimum alignment length. After having observed that between 35-45% of the sequences were unassigned with Greengenes, RDP – the Ribosomal Database Project [67] was used as reference database for this assessment (only 4% unassigned sequences). Correlations between bacterial community profiles obtained with both denoising methods and both reference databases were analyzed with STAMP [68]. [file 1471-2180-12-306-S2.pdf]

Additional file 2

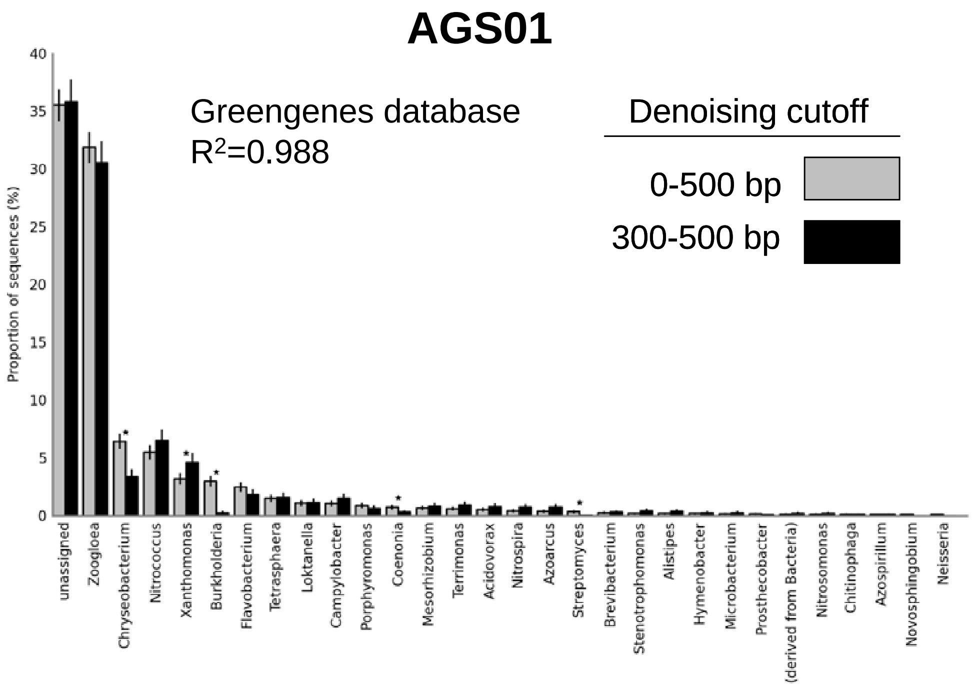

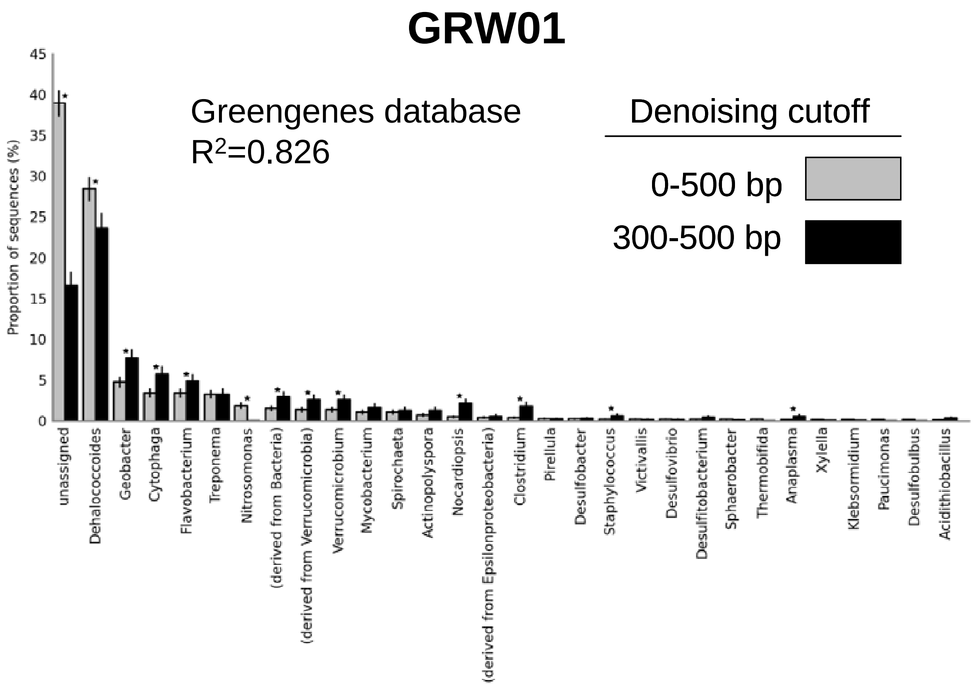

Figure AF2.1 – Comparison of bacterial community profiles obtained with the two denoising processes, after mapping with Greengenes. The first 30 predominant populations are shown.

AGS01

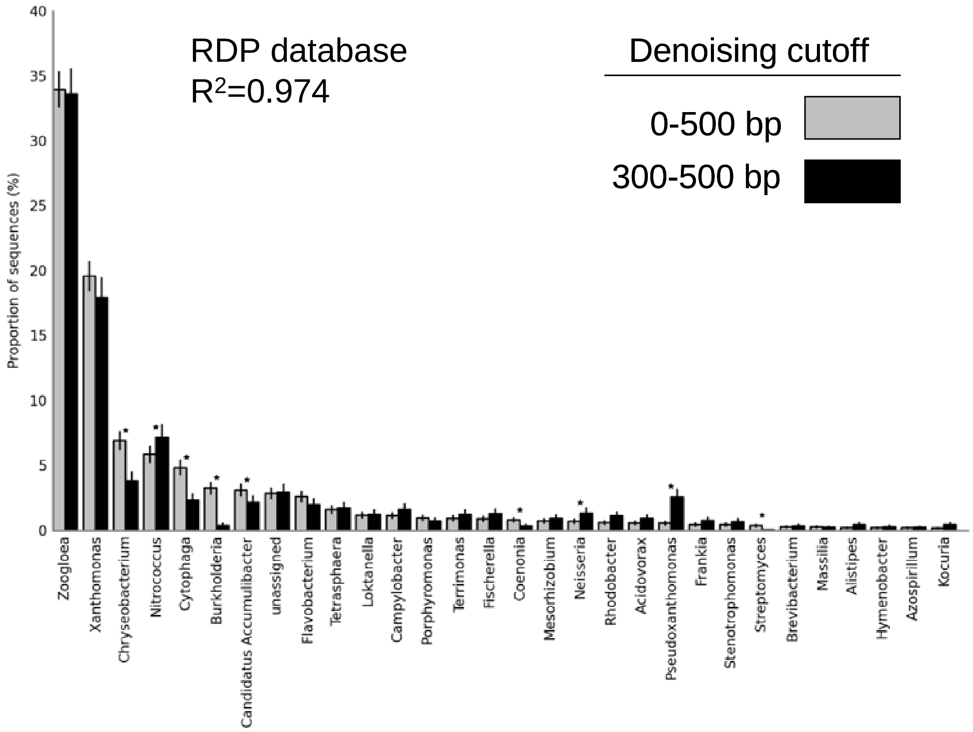

GRW01

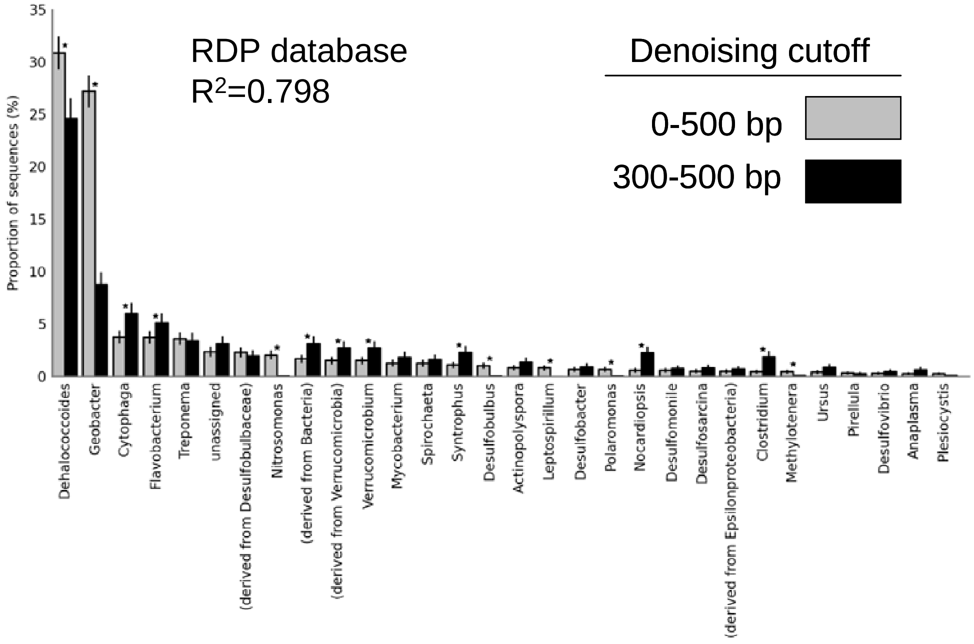

Figure AF2.2 – Comparison of bacterial community profiles obtained with the two denoising processes, after mapping with RDP. The first 30 predominant populations are shown.
