# Supplementary material for: PyroTRF-ID: a novel bioinformatics methodology for the affiliation of terminal-restriction fragments using 16S rRNA gene pyrosequencing data
Source: BMC Microbiol. 2012 Dec 27;12:306. doi: 10.1186/1471-2180-12-306 (PMC3566925; doi:10.1186/1471-2180-12-306)
Supplement: Additional file 3 — Comparison of the distributions of the SW mapping score and of the traditional identity score used by microbial ecologists in the field of environmental sciences for phylogenetic affiliation of sequences. The distributions of the absolute SW score (A) and of the SW scores normalized by the read lengths (B) obtained after mapping of 15 pyrosequencing datasets with the BWA-SW algorithm implemented in the PyroTRF-ID methodology are compared to the distribution of the identity score obtained after annotation of 10 pyrosequencing datasets with MG-RAST [66](C). Greengenes was used as annotation source in all cases. The obtained distributions are characterized by median (m), average (avg) and standard deviation values (s). [file 1471-2180-12-306-S3.pdf]

Additional file 3

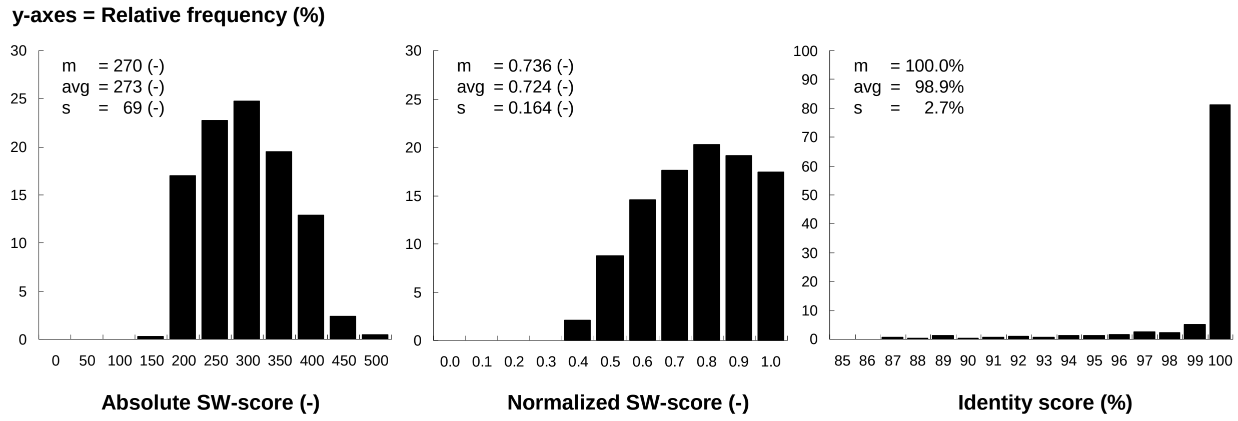

Figure AF3.1 – Comparison of the distributions of the SW mapping score and of the traditional identity score used by microbial ecologists in the field of environmental sciences for phylogenetic affiliation of sequences.

The distributions of the absolute SW score **(A)** and of the SW scores normalized by the read lengths **(B)** obtained after mapping of 15 pyrosequencing datasets with the BWA-SW algorithm implemented in the PyroTRF-ID methodology are compared to the distribution of the identity score obtained after annotation of 10 pyrosequencing datasets with MG-RAST [63] **(C)**. Greengenes was used as annotation source in all cases. The obtained distributions are characterized by median (m), average (avg) and standard deviation values (s).
