# Supplementary material for: PyroTRF-ID: a novel bioinformatics methodology for the affiliation of terminal-restriction fragments using 16S rRNA gene pyrosequencing data
Source: BMC Microbiol. 2012 Dec 27;12:306. doi: 10.1186/1471-2180-12-306 (PMC3566925; doi:10.1186/1471-2180-12-306)
Supplement: Additional file 4 — Full digital T-RFLP profiles. Examples of full digital T-RFLP profiles obtained with the restriction enzymes HaeIII and MspI for the samples GRW01 (A) and AGS01 (B). [file 1471-2180-12-306-S4.pdf]

Additional file 4

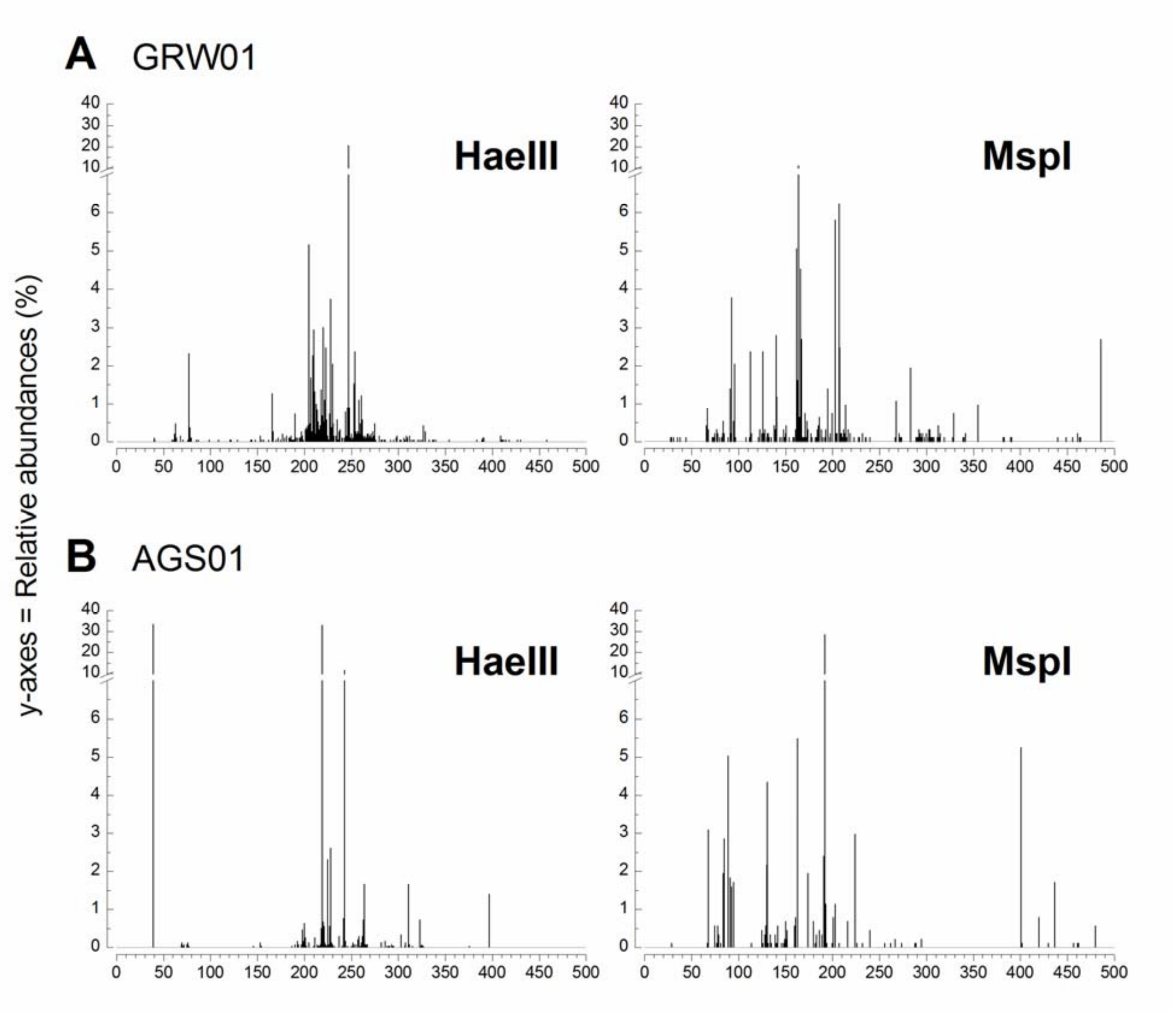

Figure AF4.1 – Examples of full digital T-RFLP profiles obtained with the restriction enzymes *Hae*III and *Msp*I for the samples GRW01 (A) **and AGS01** (B)**.**
