# Supplementary material for: PyroTRF-ID: a novel bioinformatics methodology for the affiliation of terminal-restriction fragments using 16S rRNA gene pyrosequencing data
Source: BMC Microbiol. 2012 Dec 27;12:306. doi: 10.1186/1471-2180-12-306 (PMC3566925; doi:10.1186/1471-2180-12-306)
Supplement: Additional file 5 — Comparison of mirror plots obtained on raw (left) and on denoised (right) pyrosequencing datasets. Examples are given for the sample GRW01 pyrosequenced with the HighRA method (A) and for the samples GRW07 (B) and AGS01 (C) pyrosequenced with the LowRA method. [file 1471-2180-12-306-S5.pdf]

Additional file 5

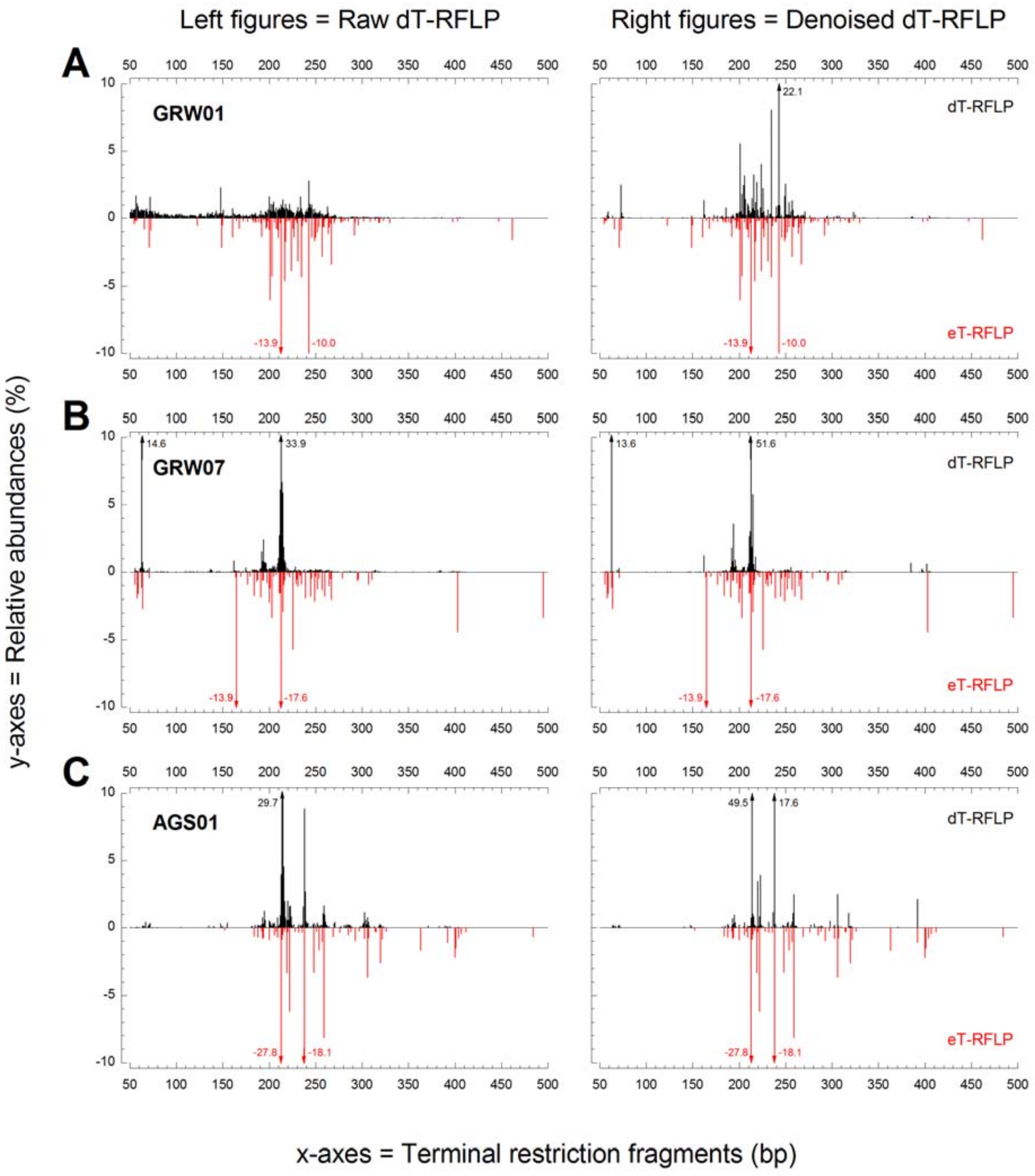

Figure AF5.1 – Comparison of mirror plots obtained on raw (left) **and on denoised** (right) **pyrosequencing datasets**

Examples are given for the sample GRW01 pyrosequenced with the HighRA method **(A)** and for the samples GRW07 **(B)** and AGS01 **(C)** pyrosequenced with the LowRA method. Relative abundances are given next to the cut predominant peaks.

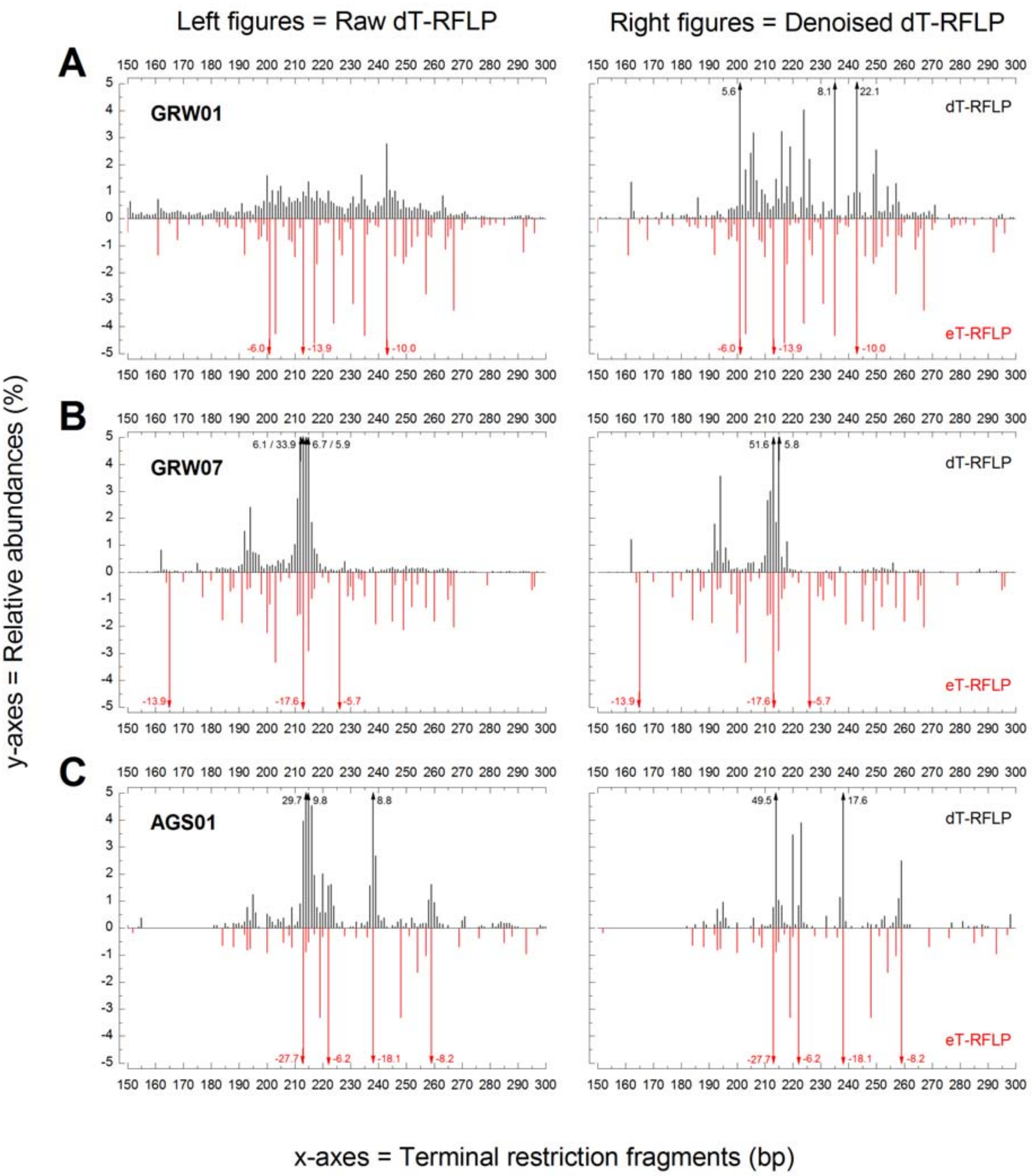

Figure AF5.2 – Comparison of mirror plots obtained on raw (left) **and on denoised** (right) **pyrosequencing datasets (zoom of Figure AF5.1 to detect low abundant peaks).**

Examples are given for the sample GRW01 pyrosequenced with the HighRA method **(A)** and for the samples GRW07 **(B)** and AGS01 **(C)** pyrosequenced with the LowRA method. Relative abundances are given next to the cut predominant peaks.
