# Supplementary material for: PyroTRF-ID: a novel bioinformatics methodology for the affiliation of terminal-restriction fragments using 16S rRNA gene pyrosequencing data
Source: BMC Microbiol. 2012 Dec 27;12:306. doi: 10.1186/1471-2180-12-306 (PMC3566925; doi:10.1186/1471-2180-12-306)
Supplement: Additional file 6 — Assessment of cross-correlation and optimal lag between denoised dT-RFLP and eT-RFLP profiles. The denoised dT-RFLP profiles of the samples AGS07 (A) and GRW04 (B) were both shifted with optimal lags of −5 bp to match with the related eT-RFLP profiles. At these optimal lags, the maximum cross-correlation coefficients amounted to 0.91 (AGS07) and 0.71 (GRW04). [file 1471-2180-12-306-S6.pdf]

Additional file 6

A

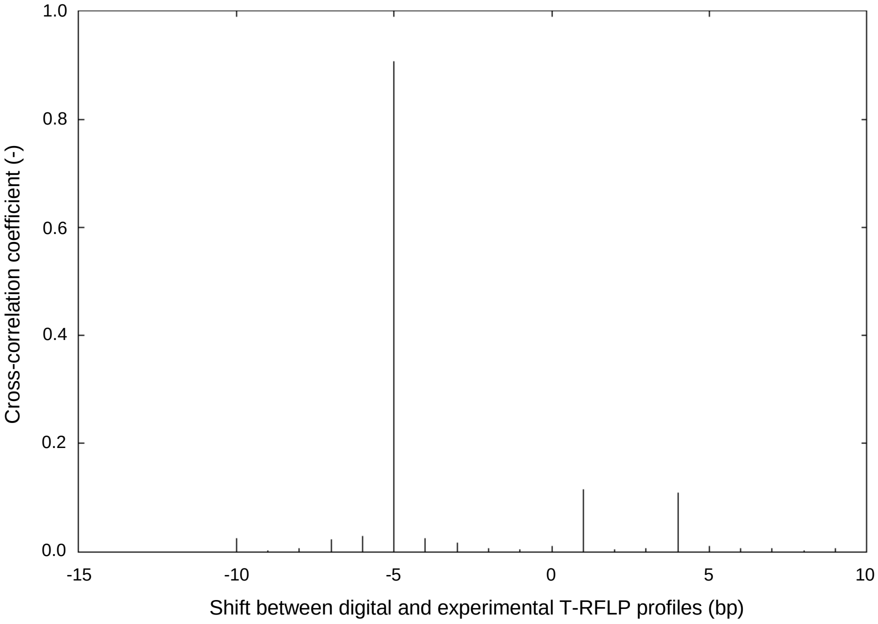

B

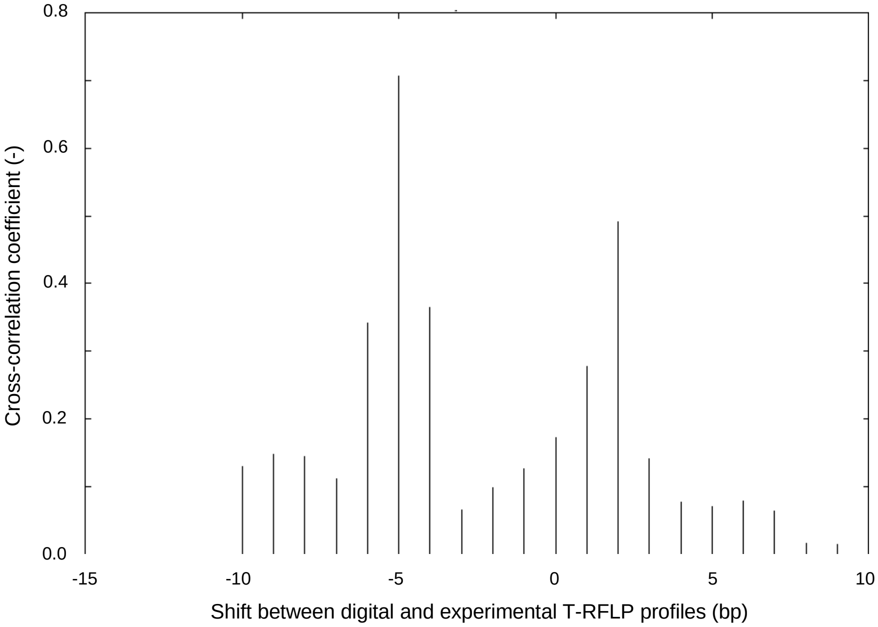

Figure AF6.1 – Assessment of cross-correlation and optimal lag between denoised dT-RFLP and eT-RFLP profiles

The denoised dT-RFLP profiles of the samples AGS07 **(A)** and GRW04 **(B)** were both shifted with optimal lags of -5 bp to match with the related eT-RFLP profiles. At these optimal lags, the maximum cross-correlation coefficients amounted to 0.91 (AGS07) and 0.71 (GRW04).
