# Supplementary material for: PyroTRF-ID: a novel bioinformatics methodology for the affiliation of terminal-restriction fragments using 16S rRNA gene pyrosequencing data
Source: BMC Microbiol. 2012 Dec 27;12:306. doi: 10.1186/1471-2180-12-306 (PMC3566925; doi:10.1186/1471-2180-12-306)
Supplement: Additional file 7 — Alignment of sequences mapping with the same reference sequence with identical accession number in the Greengenes database, and resulting in different digital T-RFs. Examples are given for the Rhodocyclus tenuis affiliates (accession number AB200295) of sample AGS01 and for Dehalococcoides relatives (accession number EF059529) of sample GRW05. [file 1471-2180-12-306-S7.pdf]

Additional file 7

```
Shifted dTRF       5|   10|   15|   20|   25|   30|   35|   40|   45|   50|   55|   60|

TRF_034      1 GATGAACGCTAGCGGCAGGCCTAATACATGCAAGTCGAACGGCAGCACGGGGG-CAACCC
TRF_194      1 ATTAAACGCTGGCGGCATGCCTTACACATGCAAATCGAACGGCAGCACGGGGG-CAACCC
TRF_213      1 ATTAAACGCTGGCGGCATGCCTTACACATGCAAGTCGAACGGCAGCACGGGGG-CAACCC
TRF_214      1 ATTAAACGCTGGCGGCATGCCTTACACATGCAAGTCGAACGGCAGCACGGGGG-CAACCC
TRF_220      1 ATTAAACGCTGGCGGCATGCCTTACACATGCAAGTCGAACGGCAGCACGGGGGGCAACCC
TRF_247      1 ATTAAACGCTGGCGGCATGCCTTACACATGCAAGTCGAACGGCAGCACGGGGG-CAACCC

TRF_034     60 TGGTGGCGAGTGGCGGACGGGTGAGTAAAGCATCGGAACGTATCCTGGAGTGGGGGA-TA
TRF_194     60 TGGTGGCGAGTGGCGGACGGGTGAGTAAAGCATCGGAACGTATCCTGGAGTGGGGGA-TA
TRF_213     60 TGGTGGCGAGTGGCGGACGGGTGAGTAAAGCATCGGAACGTATCCTGGAGTGGGGGA-TA
TRF_214     60 TGGTGGCGAGTGGCGGACGGGTGAGTAAAGCATCGGAACGTATCCTGGAGTGGGGGA-TA
TRF_220     61 TGGTGGCGAGTGGCGGACGGGTGAGTAAAGCATCGGAACGTATCCTGGAGTGGGGGAGTA
TRF_247     60 TGGTGGCGAGTGGCGGACGGGTGAGTAAAGCATCGGAACGTATCCTGGAGTGGGGGA-TA

TRF_034    119 ACGTAGCGAAAGTTA-CGCTAATACCGCATATTCTGTGAGCAGGAAAG-CAGGGGATCGC
TRF_194    119 ACGTAGCGAAAGTTA-CGCTAATACCGCATGCGCCTTCGGGGGAAAGCC--GGGACCTT
TRF_213    119 ACGTAGCGAAAGTTA-CGCTAATACCGCATATTCTGTGAGCAGGAAG--CAGGGGATCGC
TRF_214    119 ACGTAGCGAAAGTTA-CGCTAATACCGCATATTCTGTGAGCAGGAAAG-CAGGGGATCGC
TRF_220    121 ACGTAGCGAAAGTTAACGCTAATACCGCATATTCTGTGAGCAGGAAAGACAGGGAGTCGC
TRF_247    119 ACGTAGCGAAAGTTA-CGCTAATACCGCATATTCTGTGAGCAGGAAAG-CGGGGGATCGC

TRF_034    177 AAGACCTTGCGCTCT--GGGAGCGGCCGATGTCGGATTAGCTAGTTGGTGGG-TAAA--G
TRF_194    176 CGGGCCTGGCGCGAT--TGGATGAGCCCATGTCGGATTAGCTAGTTGGCGGG-TAAG---
TRF_213    176 AAGACCTTGCGCTCT--GGGAGCGGCCGATGTCGGATTAGCTAGTTGGTGGG-TAAA--G
TRF_214    177 AAGACCTTGCGCTCT--GGGAGCGGCCGATGTCGGATTAGCTAGTTGGTGGG-TAAA--G
TRF_220    181 AAGACCTTGCGTTCTTGGGAGGCGGCCGATGTCGGATTAGCTAGTTGGTGGGGTAAAACG
TRF_247    177 AAGACCTCGCGCTGT–GGGATGAGCCTACGTCCG ATTAGCTAGTTGGTGGG-TAAA--G

TRF_034    232 GCCTACCAA-GGCGACGATCCGTAGCGGGTCTGAGAGGATGATCCGCCACACTGGGACTG
TRF_194    230 GCCCACCAA-GGCGACGATCCGTAGCGGGTCTGAGAGGATGATCCGCCACACTGGGACTG
TRF_213    231 GCCTACCAA-GGCGACGATCCGTAGCGGGTCTGAGAGGACGATCAGCCACACTGGGACTG
TRF_214    232 GCCTACCAA-GGCGACGATCCGTAGCGGGTCTGAGAGGATGATCCGCCACACTGGGACTG
TRF_220    241 GCCTACCAAAGGCGACGATCCGTAGCGGGTCTGAGAGGACGATCAGCCACACT-------
TRF_247    232 GCCTACCAA-GGCGATGATCGGTAGCTGGTCTGAGAGGACGACCAGCCACACTGGGACTG

TRF_034    291 AGACACGGCCCAGACTCCTACGGGAGGCAGCAGTGGGG-AATTTTGGACAATGGGCGCAA
TRF_194    289 AGACACGGCCCAGACTCCTACGGGAGGCAGCAGTGGGA--ATTTTGGACAATGGGCGCAA
TRF_213    290 AGACACGGCCCAGACTCCTACGGGAGGCAGCAGTGGGG-AATTTTGGACAATGGGCGCAA
TRF_214    291 AGACACGGCCCAGACTCCTACGGGAGGCAGCAGTGGGG-AATTTTGGACAATGG-CGCAA
TRF_220        ------------------------------------------------------------
TRF_247    291 AGACACGGCCCAGACTCCTACGGGAGGCAGCAGTGGGGGAATATTGGACAATGGGCGCAA

TRF_034    350 GCCTGATCCAGCCATGCCGCGTGAGTGAAGAAGGCCTTCGGTTGTAA---
TRF_194    347 GCCTGATCCAGCCATGCCGCGTGAGTGAAGAAGGCCT-------------
TRF_213    349 GCCTGATCCAGCCATGCCGCGTGAGTGAAG--------------------
TRF_214    349 GCCTGATCCAGCCATGCCGCGTGAGTGAAGAAGGCCTGTCGTA-------
TRF_220        --------------------------------------------------
TRF_247    351 GCCTGATCCAGCAATGCCGCGTGGGTGAAGACGGCCTGCGGGTTGTAAAG
```

Figure AF7.1 – Alignment of sequences affiliating with *Rhodocyclus tenuis* (accession number AB200295), and exhibiting different T-RFs.

Sequence similarities are given in black. The first HaeIII-related ‘GG^CC’ restriction sites are given in red. Further restriction sites are given in yellow. The horizontal scale bare provides the number of characters per line, including alignment gaps. The bold numbers indicate the actual position in the sequence, excluding alignment gaps. The shifted dT-RF size is obtained by adding the forward primer length (20 bp) and subtracting the shift between dT-RFLP and eT-RFLP profiles (-5 bp) to the position of the first restriction site.

```
Shifted dTRF        5|   10|   15|   20|   25|   30|   35|   40|   45|   50|   55|   60|

TRF_161       1 GATG-AACGCTAGCGG-CGTGCCTTATG-CATGC-AAGTC-GAACGGTCTTAAG----CA
TRF_163       1 GATG-AACGCTAGCGG-CGTGCCTTATG-CATGC-AAGTC-GAACGGTCTTAAG----CA
TRF_164       1 GATG-AACGCTAGCGG-CGTGCCTTATG-CATGC-AAGTC-GAACGGTCTTAAG----CA
TRF_165       1 GATG-AACGCTAGCGG-CGTGCCTTATG-CATGC-AAGTC-GAACGGTCTTAAG----CA
TRF_166       1 GATG-AACGCTAGCGG-CGTGCTTTATG-CATGC-AAGTC-GAACGGTCTTAAG----CA
TRF_168       1 GATG-AACGCTAGCGG-CGTGCCTTATG-CATGC-AAGTC-GAACGGTCTTAAG----CA
TRF_171       1 GACG-AACGCTGGCGG-CGTGCCTAACA-CATGC-AAGTC-GAACGGAGATACGATTTCG
TRF_174       1 GATGTAACGCTAGCGGTCGTGCCTTATGGCATGCTAAGTCCGAACGGTCTTAAAG---CA

TRF_161      52 ATT--AAGA--TAGTGGCGAACGG-TGAGTAACGCGTAAGTAACCTACCTCTAAGTGGGG
TRF_163      52 ATT--AAGA--TAGTGGCGGACGGGTGAGTAACGCGTAAGTAACCTACCTCTAAGTGGGG
TRF_164      52 ATT--AAGA--TAGTGGCGAACGGGTGAGTAACGCGTAAGTAACCTACCTCTAAGTGGGG
TRF_165      52 ATT--AAGA--TAGTGGCGAACGGGTGAGTAACGCGTAAGTAACCTACCTCTAAGTGGGG
TRF_166      52 ATT--AAGA--TAGTGGCGAACGGGTGAGTAACGCGTAAGTAACCTACCTCTAAGTGGGG
TRF_168      52 ATT--AAGA--TAGTGGCGAACGGGTGAGTAACGCGTAAGTAACCTACCTCTAAGTGGGG
TRF_171      56 GTT--GTATCTTAGTGGCGGACGGGTGAGTAACGCGTAAGTAACCTACCTCTAAGTGGGG
TRF_174      58 ATTTAAAGATACGTTGGCGAACGGGTGAGTAACGCGTAAGTAACCTACCTCTAAGTGGGG

TRF_161     107 -A-TAGCTTCGGG-AAAC-T-GAGGGTAATA-CCGC-ATGTGGTGGGCCG-ACATATGTT
TRF_163     108 GA-TAGCTTCGGG-AAAC-T-GAAGGTAATA-CCGC-ATGTGGTGGGCCG-ACATATGTT
TRF_164     108 GA-TAGCTTCGGG-AAACGT-GAAGGTAATA-CCGC-ATGTGGTGGGCCG-ACATATGTT
TRF_165     108 GAGTAGCTTCGGG-AAACGT-AGAGGTAATA-CCGC-ATGTGGTGGGCCG-ACATATGTT
TRF_166     108 GAGTAGCTTCGGGTAAACGT-GAAGGTAATA-CCGC-ATGTGGTGGGCCG-ACATATGTT
TRF_168     108 GAGTAGCTTCGGG-AAACGTAGAAGGTAATAACCGCCATGTGGTGGGCCGGACATATGTT
TRF_171     114 GAGTAGCTTCGGG-AAACGT-GAAGGTAATA-CCGC-ATGTGGTGGGCCG-ACATATGTT
TRF_174     118 GATAGTCTTCGGG-AAAC-T-GAAGGTAATA-CCGC-ATGTGGTGGGCCG-ACATATGTT

TRF_161     159 GGTTCACCTAAAGCCGTAAGGCGCTT-GGTGAGGGGC-TTGCGTCCGATTAGCTAG-TTG
TRF_163     161 GGTTCAC-TAAAGCCGTAAGGCGCTT-GGTGAGGGGC-TTGCGTCCGATTAGCTAG-TTG
TRF_164     162 GGTTCACTAAAG-CCGTAAGGCGCTT-GGTGAGGGGC-TTGCGTCCGATTAGCTAG-TTG
TRF_165     163 GGTTCACTAAAGACCGTAAGGCGCTT-GGTGAGGGGCGTTGCGTCCGATTAGGTAGGTTG
TRF_166     164 GGTTCACTAAAGACCGTAAGGCGCTT-GGTGAGGGGCGTTGCGTCCGATTAGCTAG-TTG
TRF_168     167 GGTTCACTAAAGACCGTAAGGCGCTT-GGTGAGGGGCGTTGCGTCCGATTAGCTAG-TTG
TRF_171     169 GGTTCACTAAAG-CCGTAAGGCGCTT-GGTGAGGGGC-TTGCGTCCGATTAGCTAG-TTG
TRF_174     172 GGTTCACTAAAG-CCGTAAGGCGCTTCGGTGAGGGGGCTTGCGTCCGATTAGCTAGTTCG

TRF_161     216 GTGGGGTAA-TGGCCTACCAAGGCTT-CGATCGGTAGCTGGTCTGAGAGGATGATCAGCC
TRF_163     217 GTGGGGTAA-TGGCCTACCAAGGCTT-CGATCTGTAGCTGGTCTGAGAGGACGACCAGCC
TRF_164     218 GTGGGGTAA-CGGCCCACCAAGGCGA-CGATCGGTAGGGGGTGTGAGAGCATGACCCCCC
TRF_165     222 GTGAGGTAA-CGGCTCACCAAGCCTCGCGATCGGTAGCTGGTCTGAGAGGATGATCAGCC
TRF_166     222 GTGGGGTAA-GTGACTACCAAGGCTT-CGATCGGTAGCTGGTCTGAGAGGATGATCAGCC
TRF_168     225 GTGGGGTAAGTGGCCTACCAAGGCTT-CGATCGGTAGCTGGTCTGAGAGGACGACCAGCC
TRF_171     225 GTGGGGTAAGTGGCCTACCAAGGCTT-CGATCGGTAGCTGGTCTGAGAGGATGATCAGCC
TRF_174     231 GTGGGGTAA-TGGCCTACCAAGGCTC-CGATCGGTAGCTGGTCTGAGAGGATGATCAGCC

TRF_161     274 AC-ACTGGAAC-TGAGAC-ACGGTCCAGACTCCTACGGGAGGCAAGCAGTAAGGAA-TAT
TRF_163     275 AC-ACTGGAAC-TGAGAC-ACGGTCCAGACTCCTACGGGAGGCA-GCAGTGGGGAA-TCT
TRF_164     276 ACCACGGTACGGTCGTAG-ACGACCGGAACCCGGACCCGTACCTTACCGGGTAACGGGGA
TRF_165     281 AC-ACTGGGAC-TGAGAC-ACGGCCCAGACTCCTACGGGAGGCA-GCAGTGGGGGAATAT
TRF_166     280 AC-ACTGGGACGTGGGACGACGGCCCAGACTCCTACGGGAGGCA-GCAGCAAGGAAATCT
TRF_168     284 AC-ACTGGAAC-TGAGAC-ACGGTCCAGACTCCTACGGGAGGCA-GCAGTGGGGAAG---
TRF_171     284 AC-ACTGGGACGTGAGAC-ACGGCCC----------------------------------
TRF_174     289 ACTACTGGGAC-TGAGAC-AC---------------------------------------

TRF_161     330 TGGTCAATGGACGCAAGTCTGAACCAGCCATGCCGCGTGCAGGAA---------------
TRF_163     330 TGGACAATGGGCGCAAGCCTGATCCAGCAATGCCGCGTGAGTGATGAAGGCCTTAGGGTT
TRF_164     335 CGGACGACGACGTAAAGGGTAAAAGGTAAATTTAGGTTACGGAAACTAAGGTAGGGGACG
TRF_165     337 TGGACAATGGGC------------------------------------------------
TRF_166     338 TGGGCAATGGGCGAAAGCCTGACCCAGCAACGCCGCGTGAGGGATGAAGG----------
TRF_168         ------------------------------------------------------------
TRF_171         ------------------------------------------------------------
TRF_174         ------------------------------------------------------------

TRF_161         ------------------------------------------------------------
TRF_163     390 GTAAAGCTCTTTCGCACGCGACGATGATGACGGTAGCGTGAGAAGAAGCCCCGGCTAACT
TRF_164     395 GAACCTACC---------------------------------------------------
TRF_165         ------------------------------------------------------------
TRF_166         ------------------------------------------------------------
```

```
TRF_168       ------------------------------------------------------------
TRF_171       ------------------------------------------------------------
TRF_174       ------------------------------------------------------------

TRF_161       ----------------------
TRF_163   450 TCGTGCCAGCAGCCGCGGTAAT
TRF_164       ----------------------
TRF_165       ----------------------
TRF_166       ----------------------
TRF_168       ----------------------
TRF_171       ----------------------
TRF_174       ----------------------
```

Figure AF7.2 – Alignment of sequences affiliating with *Dehalococcoides* sp. (accession number EF059529), and exhibiting different dT-RFs.

Sequence similarities are given in black. The first HaeIII-related ‘GG^CC’ restriction sites are given in red. Further restriction sites are given in yellow. The horizontal scale bare provides the number of characters per line, including alignment gaps. The bold numbers indicate the actual position in the sequence, excluding alignment gaps. The shifted dT-RF size is obtained by adding the forward primer length (20 bp) and subtracting the shift between dT-RFLP and eT-RFLP profiles (-5 bp) to the position of the first restriction site.
